# Robust Heat Shock Response in *Chlamydia* Lacking a Typical Heat Shock Sigma Factor

**DOI:** 10.3389/fmicb.2021.812448

**Published:** 2022-01-03

**Authors:** Yehong Huang, Wurihan Wurihan, Bin Lu, Yi Zou, Yuxuan Wang, Korri Weldon, Joseph D. Fondell, Zhao Lai, Xiang Wu, Huizhou Fan

**Affiliations:** ^1^Department of Parasitology, Xiangya School of Basic Medicine, Central South University, Changsha, China; ^2^Department of Pharmacology, Robert Wood Johnson Medical School, Rutgers University, Piscataway, NJ, United States; ^3^Greehey Children’s Cancer Research Institute, University of Texas Health San Antonio, San Antonio, TX, United States; ^4^Department of Molecular Medicine, University of Texas Health San Antonio, San Antonio, TX, United States

**Keywords:** *Chlamydia*, heat shock response, stress response, transcriptome, transcriptional regulatory network, sigma factor, heat-induced transcription repressor, HrcA

## Abstract

Cells reprogram their transcriptome in response to stress, such as heat shock. In free-living bacteria, the transcriptomic reprogramming is mediated by increased DNA-binding activity of heat shock sigma factors and activation of genes normally repressed by heat-induced transcription factors. In this study, we performed transcriptomic analyses to investigate heat shock response in the obligate intracellular bacterium *Chlamydia trachomatis*, whose genome encodes only three sigma factors and a single heat-induced transcription factor. Nearly one-third of *C. trachomatis* genes showed statistically significant (≥1.5-fold) expression changes 30 min after shifting from 37 to 45°C. Notably, chromosomal genes encoding chaperones, energy metabolism enzymes, type III secretion proteins, as well as most plasmid-encoded genes, were differentially upregulated. In contrast, genes with functions in protein synthesis were disproportionately downregulated. These findings suggest that facilitating protein folding, increasing energy production, manipulating host activities, upregulating plasmid-encoded gene expression, and decreasing general protein synthesis helps facilitate *C. trachomatis* survival under stress. In addition to relieving negative regulation by the heat-inducible transcriptional repressor HrcA, heat shock upregulated the chlamydial primary sigma factor σ^66^ and an alternative sigma factor σ^28^. Interestingly, we show for the first time that heat shock downregulates the other alternative sigma factor σ^54^ in a bacterium. Downregulation of σ^54^ was accompanied by increased expression of the σ^54^ RNA polymerase activator AtoC, thus suggesting a unique regulatory mechanism for reestablishing normal expression of select σ^54^ target genes. Taken together, our findings reveal that *C. trachomatis* utilizes multiple novel survival strategies to cope with environmental stress and even to replicate. Future strategies that can specifically target and disrupt *Chlamydia*’s heat shock response will likely be of therapeutic value.

## Introduction

In response to adverse environmental conditions, living cells activate certain genes and repress others in order to survive and thrive. For bacteria, a variety of environmental insults, such as temperature variations, osmotic changes, antibiotics, solvents, and host immune response, can all elicit a stress response. Among these, sudden temperature increase is the most widely used model for studying the impact of stress (for reviews see Yura et al., 1993; Wu, 1995; Hecker et al., 1996; Roncarati and Scarlato, 2017).

Research on stress response has mainly focused on the mechanisms of gene activation. In bacteria, heat shock genes can be activated through two mechanisms in response to stress signals (Yura et al., 1993; Wu, 1995; Hecker et al., 1996; Narberhaus, 1999; Roncarati and Scarlato, 2017). Positive transcriptional regulation is achieved by specific alternative sigma factors of the RNA polymerase (e.g., σ^32^ in the Gram-negative *Escherichia coli* and σ^B^ in the Gram-positive *Bacillus subtilis*; Neidhardt and VanBogelen, 1981; Yamamori and Yura, 1982; Grossman et al., 1984; Völker et al., 1994; Yura, 1996; Hughes and Mathee, 1998). These sigma factors (i.e., heat shock sigma factors) guide the polymerase to the promoters of heat shock genes (Haldenwang, 1995; Yura, 1996; Helmann, 1999; Mooney et al., 2005; Feklistov et al., 2014; Rodriguez Ayala et al., 2020). Alternatively, alleviation of negative regulation can occur following heat-induced dissociation of transcriptional repressors from promoters of heat shock genes (Schulz and Schumann, 1996; Baldini et al., 1998; Narberhaus, 1999; Wilson and Tan, 2002; Hu et al., 2007; Roncarati et al., 2019). The most widely distributed transcriptional repressor controlling heat shock response in bacteria is the heat-inducible HrcA (Schulz and Schumann, 1996; Baldini et al., 1998; Wilson and Tan, 2002; Hu et al., 2007; Roncarati et al., 2019). Some bacteria encode additional transcriptional repressors, such as HspR and/or CtsR (Derre et al., 1999a,b, 2000; Narberhaus, 1999; Nair et al., 2000; Stewart et al., 2002; Spohn et al., 2004; Holmes et al., 2010; Pepe et al., 2018; Roncarati and Scarlato, 2018). Both HrcA and HspR negatively regulate the expression of a limited set of molecular chaperones including the Hsp70 DnaK and its cochaperone GrpE, and the Hsp60 GroEL (*aka* chaperonin) and its cochaperone GroES (Yura et al., 1993; Wu, 1995; Hecker et al., 1996; Schulz and Schumann, 1996; Baldini et al., 1998; Narberhaus, 1999; Stewart et al., 2002; Wilson and Tan, 2002; Spohn et al., 2004; Hu et al., 2007; Holmes et al., 2010; Roncarati and Scarlato, 2017, 2018; Pepe et al., 2018; Roncarati et al., 2019). Chaperones maintain numerous proteins (including HrcA and HspR) in their functional conformation, whereas stress, particularly heat shock and solvents, cause protein denaturation. As a result of heat shock, protein chaperones are titrated away from HrcA and HspR, thus leading to their functional loss and consequent activation of their target genes (Yura et al., 1993; Wu, 1995; Hecker et al., 1996; Narberhaus, 1999; Roncarati and Scarlato, 2017). Protein chaperones may also target some cellular proteins, such as σ^32^ and σ^H^, for degradation under normal growth conditions whereas heat shock frees the sigma factors to activate their target genes (Herman et al., 1995; Lund, 2001).

*Chlamydia* is a small Gram-negative bacterium that replicates strictly inside eukaryotic host cells. *Chlamydia* has a unique infectious cycle characterized by two cellular forms (Abdelrahman and Belland, 2005). The infectious but non-dividing elementary body (EB) invades host cells through receptor-mediated endocytosis. Within vacuoles termed inclusions, EBs convert into the proliferative but noninfectious reticulate bodies (RB). As RBs amass inside the inclusion, they differentiate back into EBs, which exit the cells (Hybiske and Stephens, 2007a,b).

The chlamydial developmental cycle leading to formation of progeny EBs can be disrupted by various stress conditions, such as interferon-γ, iron starvation, and antibiotic treatment (Belland et al., 2003; Huston et al., 2008; Xue et al., 2017; Brinkworth et al., 2018; Slade et al., 2019; Yang et al., 2020; Brockett and Liechti, 2021). As a result, chlamydiae enter a state referred to as persistence. During persistence, RBs continue to grow but fail to divide or differentiate into EBs, resulting in abnormally large cells referred to as aberrant bodies (Belland et al., 2003; Huston et al., 2008; Xue et al., 2017; Brinkworth et al., 2018; Slade et al., 2019; Yang et al., 2020; Brockett and Liechti, 2021; Shima et al., 2021). Upon return to favorable conditions, aberrant bodies convert to RBs, which resume the normal developmental cycle. Chlamydial persistence and recovery represent a significant clinical problem.

Given that chlamydial infections can cause fever in the host (Luger, 1948; Qvigstad et al., 1982; Dan et al., 1987; Wu et al., 2000; Reinhold et al., 2008, 2012; Stoner and Cohen, 2015; Clemmons et al., 2019), heat shock responses in *Chlamydia* cells have long been suspected. Indeed, Engel et al. (1990) first demonstrated increased mRNA and protein levels of GrpE and DnaK in *Chlamydia muridarum* cultures shortly after they were incubated at 45°C, whereas another work demonstrated heat shock-induced persistence in *Chlamydia trachomatis* (Huston et al., 2008). However, apart from recent studies focusing on transcriptional regulation by HrcA (Tan et al., 1996; Wilson and Tan, 2002, 2004; Wilson et al., 2005; Chen et al., 2011; Hanson and Tan, 2015), relatively little is known about the molecular mechanisms of heat shock response in *Chlamydia*.

We were interested in examining the full spectrum of the chlamydial response to heat shock, particularly in light of the fact that the pathogen encodes only three sigma factors (Stephens et al., 1998; Thomson et al., 2008). Its primary or housekeeping sigma factor σ^66^ is the counterpart of σ^70^ of *E. coli* (Engel and Ganem, 1990), while its two alternative sigma factors, σ^28^ and σ^54^, have counterparts with the same names in *E. coli* (Stephens et al., 1998; Thomson et al., 2008). Interestingly, we found that a 30 min incubation of *C. trachomatis* at 45°C upregulated the expression of both σ^66^ and σ^28^ and *hrcA*. The upregulations of these transcription regulators are likely responsible for the increased expression of at least 15.4% of *C. trachomatis* genes (judged by ≥1.5-fold increase, *p* < 0.005). For the first time, we document concurrent σ^54^ downregulation and *atoC* upregulation in a bacterium in response to heat shock. The discordant expression changes between σ^54^ and its activator *atoC* suggest a novel mechanism for fine-tuning the expression of σ^54^ target genes in response to stress.

## Materials and Methods

### Host Cells, *Chlamydia*, and Culture Conditions

Mouse L929 fibroblasts were used as host cells for *C. trachomatis*. Cells were grown as monolayer cultures at 37°C with air containing 5% CO_2_ using Dulbecco’s modified Eagle’s medium (DMEM) containing 4.5 g/L glucose and 0.11 g/L sodium pyruvate (Sigma Millipore) supplemented with fetal bovine serum (FBS; Omega Scientific) and gentamicin [final concentrations: 5% (vol/vol) and 10 μg/ml, respectively]. *Chlamydia trachomatis* L2 (strain 434/BU) was originally purchased from ATCC (Balakrishnan et al., 2006). CtL2/RFP was generated by transforming 434/BU with the pTRL2 (Δgfp) plasmid (Wurihan et al., 2020, 2021a), which carries a far-red fluorescence protein-encoding mKate gene downstream of a *C. trachomatis* promoter (Wickstrum et al., 2013).

### *Chlamydia trachomatis* Heat Shock and Recovery

For heat shock experiments, EBs were purified *via* MD-76 gradient ultracentrifugation as described previously (Caldwell et al., 1981). Near-confluent L929 cells grown on 6-well plates were inoculated with EBs at a multiplicity of infection (MOI) of one inclusion-forming unit per cell. The plates were subjected to 20 min centrifugation at 900 × *g* at RT to synchronize the infection (Wurihan et al., 2021b). Following washes with Hank’s balanced salt saline, the infected cells were cultured in the above medium containing 1 μg/ml cycloheximide in a 37°C incubator. To determine effects of heat shock on the *C. trachomatis* transcriptome, at 15.5 h postinoculation, a plate with triplicate cultures of wild-type *C. trachomatis* was transferred into a 45°C incubator and incubated for 30 min, while the control plate was kept in the 37°C incubator. Cultures were terminated at 16 h postinoculation. To determine effects of heat shock on chlamydial growth, heat shock of wild-type *C. trachomatis*- or CtL2/RFP-infected cultures was initiated at 16 h postinoculation. After incubation at 45°C for 2 to 8 h or re-incubation at 37°C for an additional 6 h, CtL2/RFP cultures were imaged under an Olympus IX51 microscope. Inclusion areas and RFP intensities were quantified using the ImageJ software as previously described (Wurihan et al., 2021a,b). Wild-type *C. trachomatis*-infected cultures were terminated by removal of the culture medium. Cells were collected into a 1.0 ml of 0.85% NaCl. Following centrifugation, the cell pellet was dissolved with 100 μl of 25 mM NaOH. The lysate was incubated at 95°C for 15 min and subsequently neutralized with 100 μl of 40 mM Tris-HCl (pH 7.2). Samples of the neutralized lysate were used as PCR template using the Applied Biosystems PowerUp SYBR Green Master Mix. Thermo Fisher QS5 qPCR machine was used for qPCR analyses for quantifying the relative copy number of the *C. trachomatis* genome (Wurihan et al., 2021b).

### Cellular RNA Isolation

Total host RNA and chlamydial RNA were isolated using TRI reagent (Millipore Sigma). DNA decontamination was achieved by using RNase-free DNase I (New England Biolabs). RNA concentration was determined using Qubit RNA HS assay kits (Thermo Fisher). Aliquots of the DNA-free RNA samples were stored at −80°C.

### RNA Sequencing and Analyses

RNA-Seq was performed as described recently (Wurihan et al., 2021b). Briefly, total RNA integrity was determined using Fragment Analyzer (Agilent) prior to RNA-Seq library preparation. Illumina MRZE706 Ribo-Zero Gold Epidemiology rRNA Removal kit was used to remove mouse and chlamydial rRNAs. Oligo(dT) beads were used to remove mouse mRNA. RNA-Seq libraries were prepared using Illumina TruSeq stranded mRNA-Seq sample preparation protocol, subjected to quantification process, pooled for cBot amplification, and sequenced with Illumina HiSeq 3000 platform with 50 bp single-read sequencing module. Short read sequences were first aligned to the CtL2 chromosome (GenBank accession # NC_010287.1) using TopHat2 aligner and then quantified for gene expression by HTSeq to obtain raw read counts per gene, and then converted to FPKM (Fragment Per Kilobase of gene length per Million reads of the library; Anders and Huber, 2010; Trapnell et al., 2012; Anders et al., 2015). DESeq, an R package commonly used for analysis of data from RNA-Seq studies and test for differential expression, was used to normalize data and find group-pairwise differential gene expression based on three criteria: *p* < 0.05, average FPKM >1, and fold change ≥1.

### Quantitative Reverse Transcription PCR

Quantitative reverse transcription PCR (qRT-PCR) was performed using the Luna Universal One-Step RT-qPCR kit (NEB, Cat. # E3005E) following manufacturer’s instructions. For each reaction with the exception of 23S rRNA qRT-PCR reactions, 10 ng of purified total host and bacterial RNA was used as initial template for cDNA synthesis. For quantifying 23 rRNA, 10 pg. of RNA was used for each reaction. All RT-qPCR reactions were performed in technical duplicate or triplicate. Thermo Fisher QS5 qPCR machine was used for qRT-PCR analyses.

### Functional Classification of Genes

COG functional classification of the *C. trachomatis* proteome (Galperin et al., 2015) was performed with modifications described in previous publications (Wu et al., 2011; Wurihan et al., 2021b) or community-developed *Chlamydia* databases, namely, Chlambase (Putman et al., 2019) and Chlamdb (Pillonel et al., 2020) perform.

### Controlling Inverted Repeat of Chaperone Expression Element and σ^28^ Promoter Search

The FIMO (find individual motif occurrences) program (Grant et al., 2011) was used to search for controlling inverted repeat of chaperone expression (CIRCE) elements and σ^28^ promoters in the *C. trachomatis* genome. The reference motifs were the modified CIRCE element sequence (TAGCA-N15-TGCTAA) identified by De Barsy et al. (2016) and the consensus σ^28^ promoter binding sequence identified by Yu et al. (TAAAGWWY-N11/12-RYCGAWRN). The search was restricted to 500 nucleotides upstream of the predicted translation start sites for all genes.

### Transcriptional Regulatory Network Development

The heat shock transcriptional regulatory network (TRN) was developed for significantly differentially regulated genes (i.e., genes with a ≥1.5-fold change, *p* < 0.005). Previously identified physical and/or functional associations were automatically integrated into the heat shock TRN using STRING v11 (Szklarczyk et al., 2018). The STRING v11 network was exported to Gephi (Bastian et al., 2009) on which associations identified based on the literature and/or experimental findings from this study were manually developed.

### Statistical Analysis

Inclusion area and RFP intensity, and qRT-PCR data were analyzed using *t*-tests in Excel of Microsoft Office. When applicable, values of *p* were adjusted for multiple comparisons by Benjamini-Hochberg procedure to control the false discovery rate (Benjamini and Hochberg, 1995).

## Results

### Heat Shock Induces Robust Transcriptomic Changes in *Chlamydia trachomatis*

**Figure 1:**
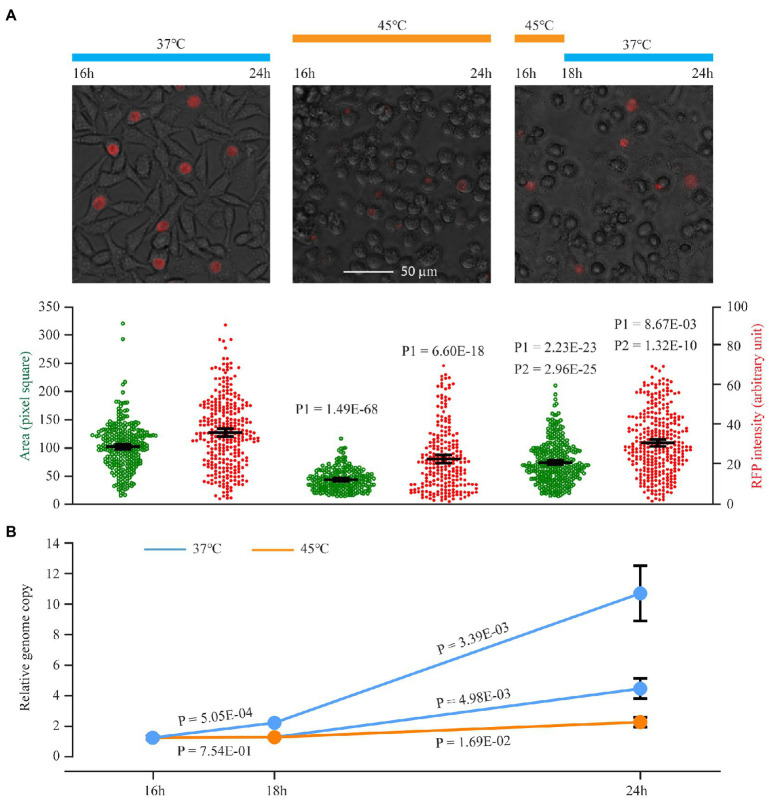
An initial *C. trachomatis* growth arrest following a switch to 45°C, adaptation upon continued incubation at 45°C, and growth recovery after return to 37°C. **(A)** Expansion of inclusion size and increased plasmid-expressed RFP intensity upon recovery from heat shock at 45°C. *Chlamydia trachomatis* CtL2/RFP-infected cells were cultured at 37°C. At 16 h postinoculation, cultures were left at 37°C (left) or subjected to heat shock at 45°C for 8 h (center) or 2 h followed by a 6 h recovery at 37°C (right). At 24 h, RFP and white light phase-contrast images were acquired. The culture temperatures during the experiments are shown on top, representative cell images in the middle, and quantitative inclusion areas and RFP intensities (averages ±95% confidential intervals) at the bottom. The scale bar is applicable to all three images. P1 indicates values of *p* of *t*-tests between heat-shocked cultures and control cultures. P2 indicates values of *p* of *t*-tests between cultures incubated at 45°C for 2 h followed by a 6 h recovery at 37°C and cultures incubated at 45°C for 8 h (i.e., no recovery). **(B)** Genome replication in *C. trachomatis* cultured at 45°C. Wild-type *C. trachomatis* were subjected to heat shock as in **(A)**. Relative genome copy numbers were quantified using qPCR. Data represent averages ± standard deviations of biological triplicates.

To obtain a snapshot of *C. trachomatis* heat shock response at the transcriptomic level, we performed RNA sequencing (RNA-Seq) analyses and compared the transcriptome of a set of triplicate *C. trachomatis* cultures incubated at 45°C for 30 min with that of a set of control cultures maintained at the routine culture temperature 37°C (Figure 1). We chose these experimental settings to determine the full capacity of transcriptomic reprogramming in response to heat shock, even though *C. trachomatis* infection almost certainly does not increase the body temperature to such a degree. Previous heat shock studies with *C. trachomatis* and *C. muridarum* were performed under similar conditions (Engel et al., 1990; Karunakaran et al., 2003; Hanson and Tan, 2015). Importantly, *C. trachomatis* can recover from heat shock at 45°C. As shown in Figure 1A, red fluorescence protein-expressing *C. trachomatis* cultures incubated at 45°C for 2 h formed statistically significantly larger inclusions with more intense RFP signals following a 6 h recovery at 37°C, compared to cultures maintained at 45°C without the recovery (Figure 1A). Likewise, quantitative PCR analysis detected a higher level of the bacterial genome in wild-type *C. trachomatis* cultures exposed to a 2-h incubation at 45°C followed by a 6 h recovery at 37°C relative to heat-shocked cultures without the recovery (Figure 1B). Interestingly, it appears that *C. trachomatis* partially regained the capacity to replicate its genome when cultured at 45°C for an extended period following an initial complete halt of genome replication. The genome-doubling time at 45°C is estimated to be approximately 6 h, which is three times longer than the normal 2 h (Figure 1B).

Following incubation at 45°C for 30 min, 303 (30.9%) of the total 979 genes in the *C. trachomatis* genome underwent ≥1.50-fold expression changes with values of *p* < 0.005. Transcript copy numbers of 151 (15.4%) genes increased (Supplementary Table S1), while those of 152 (15.5%) genes decreased (Supplementary Table S2). These findings suggest that *C. trachomatis* is capable of mounting a robust heat shock response *via* transcriptomic reprogramming even though it lacks a typical heat shock sigma factor.

**Figure 2:**
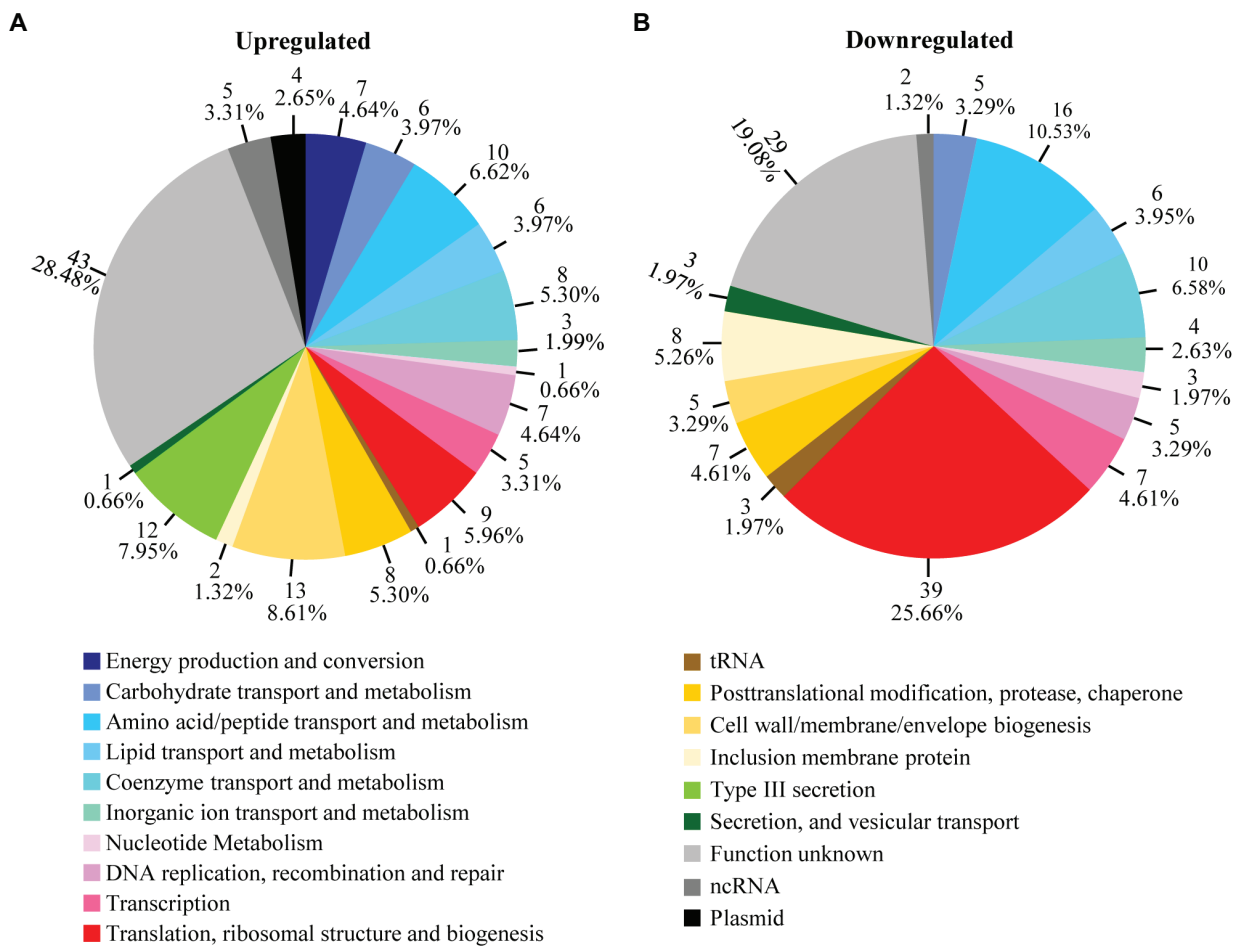
Heat shock-regulated genes in functional groups. Transcripts that were upregulated **(A)** or downregulated **(B)** by ≥1.5-fold (*p* < 0.005) as determined by RNA-Seq following culture at 45°C between 15.5 and 16.0 h postinoculation are organized in pie charts by their functional groups. Shown adjacently to each pie slice is the number of genes and the percentages of up- or downregulated genes in the group. See Supplementary Tables S1 and S2 for identities and functions of individual genes in the functional groups.

When the differentially regulated genes were placed into functional groups, it became apparent that certain functional gene groups are disproportionately regulated by heat shock (Figure 2A). While genes involved in energy metabolism, posttranslational modification, protein turnover, type III secretion, and plasmid-encoded genes are disproportionately upregulated (Figure 2A), genes involved in amino acid and peptide transport and metabolism and ribosomal structure and biogenesis are disproportionately downregulated (Figure 2B). As detailed below, quantitative reverse transcription PCR (qRT-PCR) analyses were performed to validate RNA-Seq changes, while pathway analyses were performed to reveal the effects of heat shock on distinct physiological functions and to elucidate the underlying regulatory mechanisms in *C. trachomatis*.

### Heat Shock Upregulates Energy Production and Conversion

**Figure 3:**
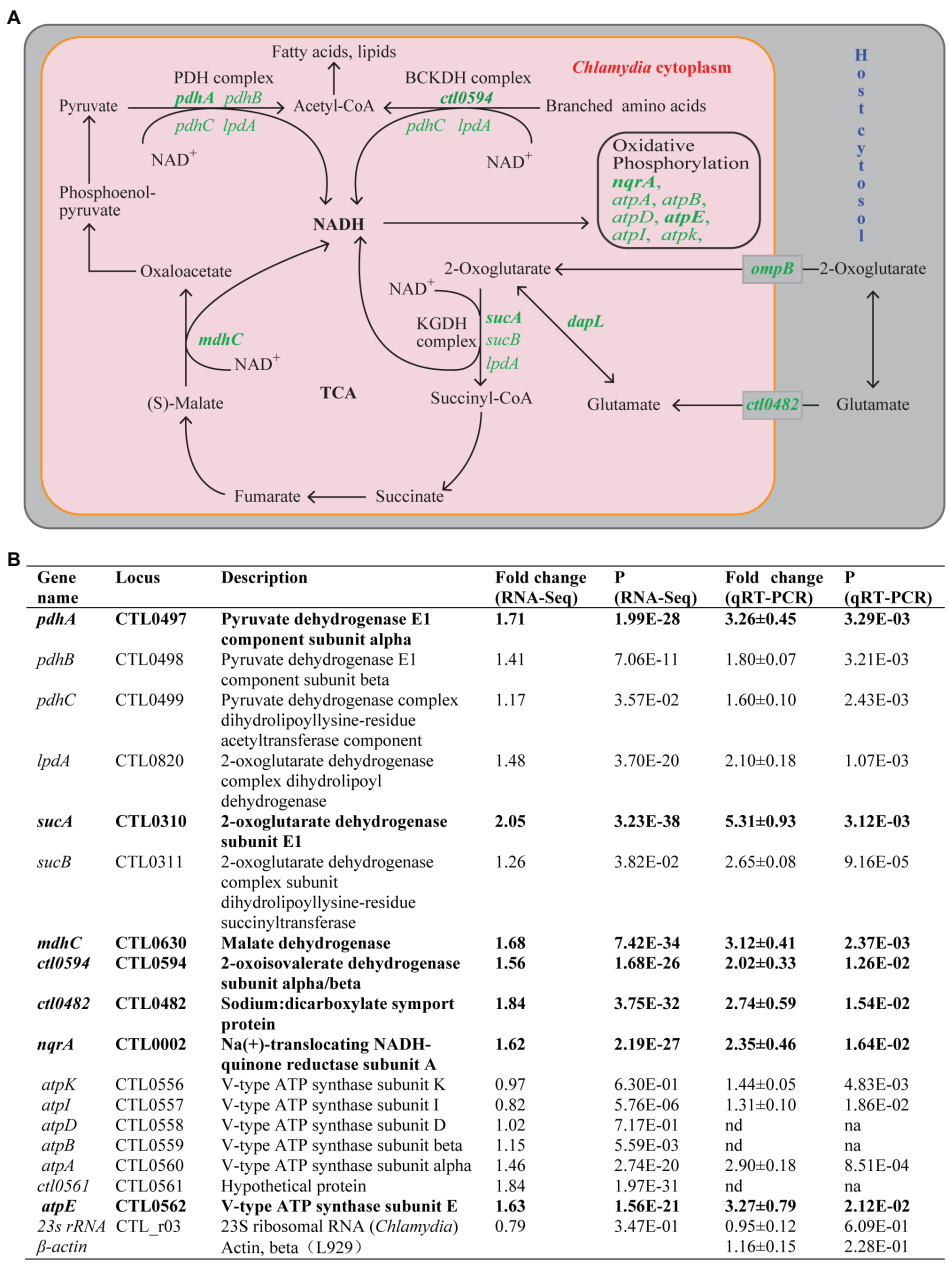
Upregulation of energy metabolism genes in response to heat shock. **(A)** Energy production and conversion pathways in *C. trachomatis*. Genes upregulated by heat shock are shown in green. Pink and gray rectangles depict chlamydial and host cytosols, respectively. **(B)** Functions and expression data of genes with demonstrated or presumptive expression increases. **(A,B)** Genes that displayed ≥1.5-fold upregulation (*p* < 0.005) in RNA-Seq are shown in bold text, while those that did not meet the upregulation criterion in RNA-Seq but displayed expression increases in qRT-PCR analysis or presumed to be increased are shown in normal text. *atpB* and *atpD* (as well as *ctl0561*) are presumed to be upregulated by heat shock (even though their expression was not analyzed by qRT-PCR) because they are located within the seven gene operon whose other four genes were significantly upregulated. **(B)** Mature 23S rRNA and host β-actin were analyzed as controls. Abbreviations: nd, not determined; na, not applicable.

Seven (4.6%) of the 151 genes with increased expression, but none of the genes with decreased expression, were in the functional group of energy metabolism. Four of these seven genes are involved in NADH production. Of these four, *pdhA* encodes the alpha subunit of the E1 component of the pyruvate dehydrogenase that produces NADH while oxidizing pyruvate to acetyl-coenzyme A (CoA), whereas *sucA* (2-oxoglutarate dehydrogenase subunit E1) and *mdhC* (malate dehydrogenase) are involved in generating NADH *via* the incomplete tricarboxylic acid (TCA) cycle (Stephens et al., 1998; Iliffe-Lee and McClarty, 1999; Figure 3A). *ctl0594* (2-oxoisovalerate dehydrogenase subunit alpha/beta) participates in the catalysis of NADH production in the branching amino acid catabolic pathway (Figure 3A). Among the three remaining upregulated genes involved in energy metabolism, *ctl0482* (sodium:dicarboxylate symport protein) facilitates the uptake of host-derived glutamate that feeds into the TCA cycle (Figure 3A), while *nqrA* (Na^+^-translocating NADH-quinone reductase subunit A) and *atpE* (V-type ATP synthase subunit E) are involved in reactions in the oxidative phosphorylation chain that produces ATP (Figure 3A).

We performed qRT-PCR analyses to validate the expression changes detected by RNA-Seq for the seven aforementioned upregulated energy metabolism-related genes. We included cotranscribed genes in the same operon of these genes in our qRT-PCR analysis. Importantly, we observed expression increases of all three subunits of the pyruvate dehydrogenase (*pdhA*, *pdhB*, and *pdhC*; Figure 3B), as well as both components of the 2-oxoglutarate dehydrogenase complex (*sucA* and *sucB*; Figure 3B). Additionally, three genes from the V-ATP synthase operon (*atpA*, *atpI*, and *atpK*) cotranscribed with the aforementioned *atpE* gene showed expression increases despite the fact that RNA-Seq detected only small or no expression increases for these genes. Although qRT-PCR was not performed for the genes *atpD* and *atpB* located in the middle of the V-ATPase operon, our findings predict the expression of these genes was likewise upregulated. Our qRT-PCR analysis also confirmed expression increases of four non-operon genes functionally involved in energy metabolism: *mdhC, nqrA, ct0594*, and *ctl0482* (Figure 3B). In addition, qRT-PCR detected a 2.1-fold expression increase of *lpdA* whose expression increase in RNA-Seq was slightly below the 1.5-fold change threshold in RNA-Seq (Figure 3B; Supplementary Table S1). LpdA participates in multiple NADH-producing reactions (Figure 3A). As controls, we performed qRT-PCR for *C. trachomatis* 23S rRNA and host mouse actin RNA; neither was changed by heat shock (Figure 3B). In summary, at least 16 energy production and conversion genes were upregulated thus suggesting that in response to heat shock, *C. trachomatis* increases its energy production.

In addition to the above “core” energy metabolic genes, RNA-Seq results also showed a 2-fold increase in *ompB* in response to heat shock (Supplementary Table S1)*. ompB* encodes a porin PorB, which mediates the acquisition of 2-oxoglutarate from the host cell (Kubo and Stephens, 2001; Figure 3A). Furthermore, RNA-Seq also showed a 2.1-fold increase of *dapL* whose gene product L, L-diaminopimelate aminotransferase converts glutamate to 2-oxoglutarate. Together, these gene upregulations provide *C. trachomatis* with increased 2-oxoglutarate for the production of NADH.

### Heat Shock Upregulates Type III Secretion

**Figure 4:**
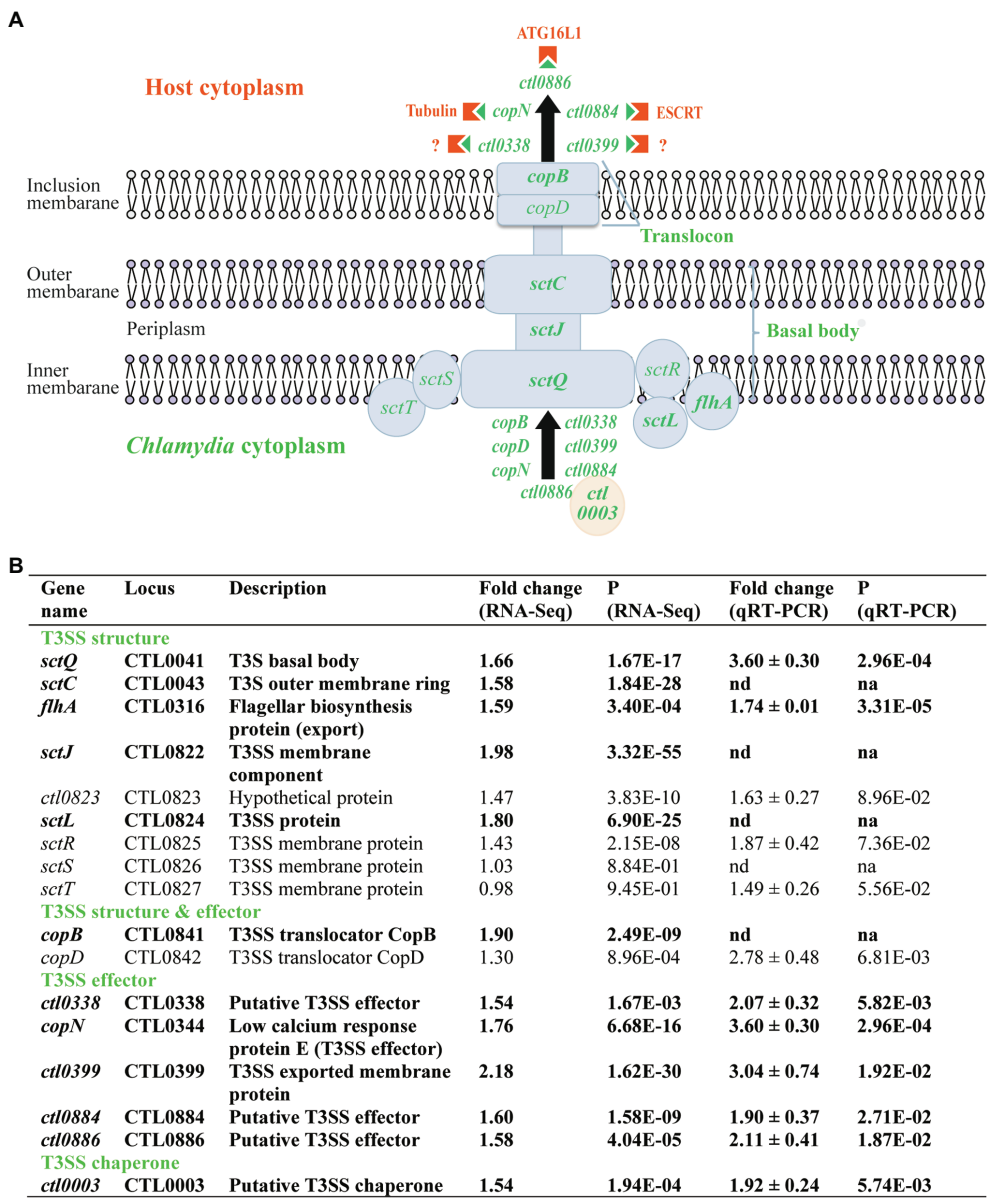
Upregulation of T3S genes in response to heat shock. **(A)** Schematic presentation of chlamydial T3SS, T3S effectors, and host components that interact with the T3S effectors. Only heat shock-upregulated T3SS and T3S effector genes are specified (shown in green). Host components targeted by the T3S effectors are shown in orange. **(B)** Functions and expression data of genes with expression increases. **(A,B)** Genes that displayed ≥1.5-fold upregulation (*p* < 0.005) in RNA-Seq are shown in bold text, while those that did not meet the upregulation criterion in RNA-Seq but displayed expression increases in qRT-PCR analysis or presumed to be increased are shown in normal text. Note *sctS* is presumed to be upregulated by heat shock (even though its expression was not analyzed by qRT-PCR) because it is cotranscribed in the same operon along with *sctJ, ctl0823, sctL, sctR*, and *sctT*, all of which showed increased transcripts by qRT-PCR and/or RNA-Seq at 45°C.

The *C. trachomatis* type III secretion (T3S) system (T3SS) secretes effector proteins to establish and maintain its intracellular growth niche (Fields and Hackstadt, 2000; Subtil et al., 2000; Fields et al., 2003, 2005; Clifton et al., 2004; Hefty and Stephens, 2007; Jamison and Hackstadt, 2008; Hobolt-Pedersen et al., 2009; Markham et al., 2009; Spaeth et al., 2009; Lutter et al., 2010; Chen et al., 2014; Nans et al., 2015; Bugalhão and Mota, 2019). Similar to energy metabolism genes, T3S-related genes are overly represented in the heat shock-upregulated gene group (Figure 2A; Supplementary Table S1). Twelve (7.9%) of the total 151 upregulated genes, but none of the 152 downregulated, were related to T3S (Figure 2; Supplementary Tables S1 and S2). Six of these genes encode proteins that constitute the T3SS structural apparatus (Figure 4A). While *flhA, sctC, sctJ, sctL*, and *sctQ* encode components of the basal body, *copB* and *copD* encode components of the translocon located in the inclusion membrane. CopB and CopD are also considered effector proteins since they are delivered to the inclusion membrane through the T3SS (Figure 4A). Included among the other six upregulated T3SS-related genes detected by RNA-Seq were *copN, ctl0338, ctl0399, ctl0884*, and *ctl0886*, all of which encode T3SS effectors (Figure 4A), as well as *ctl0003* which encodes a T3S chaperone that facilitates the secretion of effector proteins. While the functions of CTL0338 and CTL0399 have yet to be determined, CopN and CTL0884 interact with the host cytoskeleton protein tubulin (Archuleta et al., 2011; Nawrotek et al., 2014; Campanacci et al., 2019) and components of the endosomal sorting complexes required for transport (ESCRT; Vromman et al., 2016; Hamaoui et al., 2020), respectively. Furthermore, CTL0886 interacts with the host protein ATG16L1 and counteracts the restriction of ATG16L1 on chlamydial inclusion expansion (Hamaoui et al., 2020).

To validate the RNA-Seq findings, we performed qRT-PCR analyses for eight of the 12 upregulated T3SS genes and confirmed increases in their expression in response to heat shock (Figure 4B). We extended the qRT-PCR studies to include several T3SS operon genes that showed relatively small or even no expression increases by RNA-Seq despite the fact that their cotranscribed genes were upregulated. In the operon spanning from *ctl0822* to *ctl0827* (Hefty and Stephens, 2007), which encode mostly components of the basal body, RNA-Seq showed a 1.43-fold increase for *sctR*, whereas qRT-PCR showed a trending significant 1.87-fold increase (Figure 4B). qRT-PCR also showed a trending significant 1.49-fold increase in *sctT* expression even though RNA-Seq did not detect an expression change. Given these findings, it appears likely that the expression of *sctS*, the second to the last gene of the operon, is also upregulated in response to heat shock despite it not being included in this study (Figure 4B). Interestingly, we detected a statistically significant 2.78-fold increase in *copD* expression from the *copBD* operon, whereas the earlier RNA-Seq analysis had only measured a 1.30-fold increase (Figure 4B). In summary, at least 16 T3S-related genes were significantly upregulated in response to heat shock thus suggesting that *C. trachomatis* RBs secrete elevated levels of these distinct effector proteins to increase their chance of survival.

### Heat Shock Upregulates Five of the Eight Plasmid-Encoded Genes

**Figure 5:**
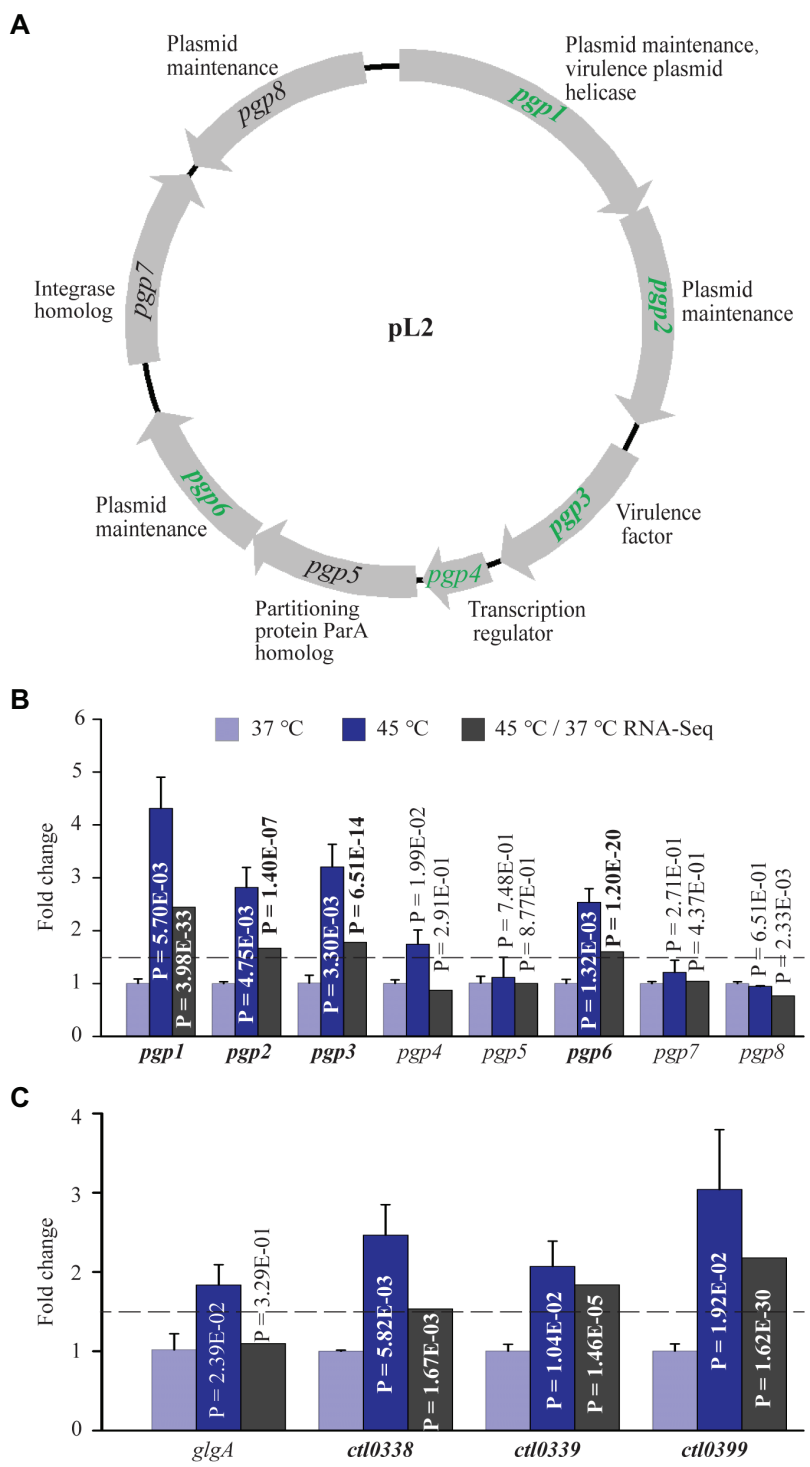
Upregulation of plasmid-encoded genes and Pgp4-regulated chromosomal genes in response to heat shock. **(A)** The pL2 plasmid map. Shown adjacently to each gene is its function. Genes upregulated by heat shock are shown in green. **(B)** qRT-PCR and RNA-Seq data for all eight plasmid-encoded genes. **(C)** Increased expression of all four Pgp4 target genes analyzed by qRT-PCR. **(A–C)** Genes that displayed ≥1.5-fold upregulation (*p* < 0.005) in RNA-Seq are shown in bold text, while those that did not meet the upregulation criterion in RNA-Seq but displayed expression increases in qRT-PCR analysis or presumed to be increased are shown in regular text.

The *C. trachomatis* plasmid encodes eight proteins (Pgp1-8) whose functions include fitness improvement (*pgp3*; Ma et al., 2020), transcriptional regulation (*pgp4* and possibly *pgp5*; Song et al., 2013; Liu et al., 2014a; Zhang et al., 2020), and plasmid maintenance (*pgp1*, *pgp2*, *pgp6*, and *pgp8*; Figure 5A). Interestingly, RNA-Seq analysis showed 2.4-, 1.67- 1.78-, and 1.60-fold upregulation for *pgp1, pgp2, pgp3*, and *pgp6*, respectively, following heat shock. qRT-PCR confirmed the upregulation of these genes (Figure 5B). In addition, qRT-PCR detected a 1.70-fold increase in *pgp4* RNA in heat-shocked cultures, even though RNA-Seq did not detect an increase. Notably, the expression of *pgp5, pgp7*, and *pgp8* were unaffected by heat shock either positively or negatively (Figure 5B).

The Pgp4 regulon is composed of *pgp3* and numerous chromosomal genes (Song et al., 2013; Patton et al., 2018; Zhang et al., 2020). To examine whether the expression of other Pgp4 regulon genes was effected by heat shock, we performed qRT-PCR for four confirmed Pgp4 chromosomal target genes including *glgA*, *ctl0338*, *ctl0339*, and *ctl0399* (Song et al., 2013; Zhang et al., 2020). RNA-Seq detected expression increases for *ctl0338, ctl0339*, and *ctl0399*, which was confirmed by qRT-PCR data (Figure 5C). Although RNA-Seq failed to detect an expression change in *glgA* following heat shock, qRT-PCR detected a 1.84-fold increase (Figure 5C). In addition, *ctl0638*, another Pgp4 target gene, was found by RNA-Seq analysis to be upregulated by 3.69-fold following heat shock (Supplementary Table S1). These findings are consistent with a role for Pgp4 in upregulation of its target genes in response to stress. Taken together, the increased expression of plasmid-encoded genes supports the notion that the *C. trachomatis* plasmid serves as an important virulence determinant that helps chlamydiae survive during heat shock.

### Heat Shock Disproportionately Downregulates Genes With Functions in Protein Synthesis

In contrast to energy metabolism, T3S, and plasmid-encoded genes, RNA-Seq revealed that genes involved in protein translation, ribosomal structure and biogenesis are disproportionately downregulated in heat-shocked *C. trachomatis* cells. More than a quarter of the 152 downregulated genes (Figure 2B), but only 6% of the 151 upregulated genes (Figure 2A), encode proteins with functions in protein synthesis. The difference in the number of ribosomal protein genes in the two categories was particularly striking: 20 were downregulated, while only two were upregulated (Table 1). We performed qRT-PCR analyses for six of the downregulated genes with different functions in protein synthesis and confirmed their decreased expression (Table 1). Given that ribosomal proteins and factors involved in protein synthesis are the most abundant proteins in the bacterial cytosol, our findings suggest that RBs reduce protein synthesis in general to conserve energy and resources in response to heat shock.

**Table 1:** Heat shock-downregulated genes associated with translation and ribosomal structure and biogenesis.

Gene name	Locus	Description	Fold change (RNA-Seq)	Fold change (qRT-PCR)	*p* (qRT-PCR)
**tRNA processing**					
*rnpA*	CTL0153	Ribonuclease P protein component	−1.65	nd^*^	na^**^
*trmD*	CTL0282	Fused tRNA (guanine-N(1)-)-methyltransferase/hypothetical protein	−2.20	nd	na
*truB*	CTL0349	tRNA pseudouridine synthase B	−1.77	nd	na
*mnmA*	CTL0539	tRNA-specific 2-thiouridylase MnmA	−1.51	nd	na
*gidA*	CTL0760	tRNA uridine 5-carboxymethylaminomethyl modification protein GidA	−1.59	nd	na
**tRNA biogenesis**					
*metG*	CTL0287	Methionine--tRNA ligase	−2.19	−(1.75 ± 0.36)	1.15E-02
*valS*	CTL0554	Valine--tRNA ligase	−2.19	nd	na
*gltX*	CTL0705	Glutamate--tRNA ligase	−1.57	nd	na
*argS*	CTL0714	Arginine--tRNA ligase	−2.46	−(2.24 ± 0.50)	1.58E-02
**Initiation**					
*infC*	CTL0205	Translation initiation factor IF-3	−1.51	nd	na
*infB*	CTL0351	Translation initiation factor IF-2	−1.98	−(1.43 ± 0.08)	5.00E-03
*ctl0138*	CTL0138	Ribosome silencing factor	−1.59	nd	na
*fmt*	CTL0792	Methionyl-tRNA formyltransferase	−2.55	−(1.84 ± 0.39)	3.33E-02
**RNA processing**					
*vacB*	CTL0654	Ribonuclease R	−1.87	nd	na
*ctl0660*	CTL0660	SpoU family rRNA methylase	−2.19	nd	na
*ctl0661*	CTL0661	SAM-dependent methyltransferase	−2.17	nd	na
*rbfA*	CTL0350	Ribosome-binding factor A	−1.89	nd	na
**Subunit assembly**					
*rpmI*	CTL0206	50S ribosomal protein L35	−1.60	nd	na
*rpsP*	CTL0281	30S ribosomal protein S16	−2.38	−(1.67 ± 0.16)	8.00E-03
*rplS*	CTL0283	50S ribosomal protein L19	−2.37	−(1.55 ± 0.15)	2.99E-03
*rpsA*	CTL0353	30S ribosomal protein S1	−2.50	nd	na
*rplL*	CTL0568	50S ribosomal protein L7/L12	−1.59	nd	na
*rplO*	CTL0773	50S ribosomal protein L15	−1.81	nd	na
*rpsE*	CTL0774	30S ribosomal protein S5	−1.60	nd	na
*rplR*	CTL0775	50S ribosomal protein L18	−1.65	nd	na
*rplF*	CTL0776	50S ribosomal protein L6	−1.64	nd	na
*rpsH*	CTL0777	30S ribosomal protein S8	−1.71	nd	na
*rplE*	CTL0778	50S ribosomal protein L5	−1.78	nd	na
*rplX*	CTL0779	50S ribosomal protein L24	−1.67	nd	na
*rplN*	CTL0780	50S ribosomal protein L14	−1.55	nd	na
*rpsC*	CTL0784	30S ribosomal protein S3	−1.52	nd	na
*rplV*	CTL0785	50S ribosomal protein L22	−1.80	nd	na
*rpsS*	CTL0786	30S ribosomal protein S19	−1.52	nd	na
*rplB*	CTL0787	50S ribosomal protein L2	−1.53	nd	na
*rplW*	CTL0788	50S ribosomal protein L23	−1.69	nd	na
*rplD*	CTL0789	50S ribosomal protein L4	−2.06	−(1.69 ± 0.30)	1.52E-01
*rplC*	CTL0790	50S ribosomal protein L3	−1.68	nd	na
**Elongation**					
*tufA*	CTL0574	Elongation factor Tu	−1.77	−(1.15 ± 0.19)	3.58E-01
**Peptide maturation**					
*map*	CTL0224	Methionine aminopeptidase	−1.73	nd	na

* *Not determined*.

** *Not applicable*.

### Heat Shock Upregulates Expression of *hrcA* and Its Known and Potential Target Genes

**Figure 6:**
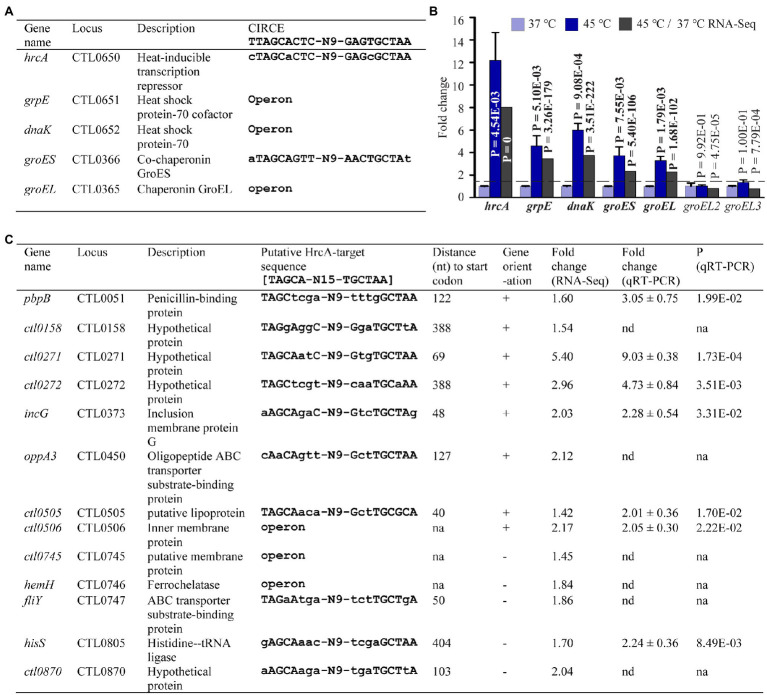
Upregulation of HrcA target and potential target genes in response to heat shock. **(A)** Name, function, and organization of five known HrcA target genes along with associated CIRCE element sequence previously shown to bind HrcA (see text for references). **(B)** qRT-PCR and RNA-Seq data for all the five known HrcA target genes. *groEL2* and *groEL3* are not target genes of HrcA. **(C)** Newly predicted potential novel HrcA target genes along with associated putative HrcA target binding sequence. Upregulated fold changes in gene expression as detected by RNA-Seq and/or qRT-PCR following heat shock are indicated.

Having validated numerous heat shock-induced transcriptomic changes, we next searched for the underling regulatory mechanisms by analyzing the expression of genes known to regulate stress response and to control gene expression in general. The *Chlamydia* genome encodes a single heat-inducible transcriptional repressor HrcA (Stephens et al., 1998; Read et al., 2000, 2003; Thomson et al., 2005) whose previously characterized regulon includes two operons (Tan et al., 1996; Wilson and Tan, 2002, 2004; Hanson and Tan, 2015). One of the HrcA-regulated operons encodes HrcA itself, the molecular chaperone DnaK (*aka* Hsp70), and its cochaperone GrpE. The other operon encodes the molecular chaperone GroEL (also known as chaperonin) and its cochaperone GroES (Figure 6A). HrcA represses transcription of these operons by binding to CIRCE elements within or near their promoters at the physiological temperature (Schulz and Schumann, 1996; Baldini et al., 1998; Narberhaus, 1999; Lund, 2001; Wilson and Tan, 2002; Hu et al., 2007; Roncarati et al., 2019). At abnormally high temperatures or under other stress conditions, HrcA loses the capacity to bind CIRCE elements, leading to activation of its target genes (Schulz and Schumann, 1996; Baldini et al., 1998; Narberhaus, 1999; Lund, 2001; Wilson and Tan, 2002; Hu et al., 2007; Roncarati et al., 2019). As expected, RNA-Seq detected elevated RNA levels of *hrcA*, *grpE*, *dnaK*, *groES*, and *groEL* (Figure 6B). For each of these HrcA target genes, qRT-PCR detected an even higher level of increased expression than that measured by RNA-Seq (Figure 6B). By contrast, neither RNA-Seq nor qRT-PCR detected changes in the expression of *groEL2* and *groEL3* (Figure 6B), which also encode protein chaperones but are not regulated by HrcA due to the absence of CIRCE elements in their promoters (Hanson and Tan, 2015).

Whereas the consensus sequence for a CIRCE element is 5′-TTAGCACTC-(N)_9_-GAGTGCTAA-3′ (Hecker et al., 1996; Narberhaus, 1999), De Barsy et al. (2016) recently proposed 5′-TAGCA-(N)_15_-TGCTAA-3′ as a *bona fide* HrcA-binding sequence based on studies with *Waddlia chondrophila*, a *Chlamydia*-like organism. Using this revised CIRCE consensus sequence, we performed motif search analyses in the *C. trachomatis* genome and identified eight non-operon and five operon genes carrying the upstream putative HrcA target sequence (Figure 6C). Interestingly, the expression of these genes increased by 1.42- to 9.03-fold as shown by RNA-Seq and/or qRT-PCR analyses (Figure 6C). Notably, these genes encode functionally diverse proteins including four hypothetical proteins (Figure 6C). Our findings thus suggest that transcriptional regulation by *C. trachomatis* HrcA not only includes chaperone-encoding genes, but genes with a variety of functions.

### Heat Shock Induces Expression Changes in All Three *Chlamydia trachomatis* Sigma Factors and Other Transcriptional Regulators

**Figure 7:**
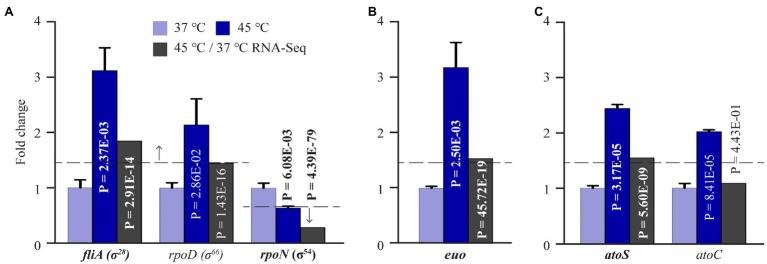
Expression changes of *C. trachomatis* transcription regulators in response to heat shock. **(A)** Increased *fliA*, *rpoD* expression and decreased *rpoN* expression as determined by both RNA-Seq and qRT-PCR. **(B)** Increased *euo* expression as determined by both RNA-Seq and qRT-PCR. **(C)** Increased *atoS* and *atoC* expression as detected by RNA-Seq and/or qRT-PCR.

HrcA-mediated transcriptional regulation accounts for only a small proportion of the heat shock-induced upregulated genes in *C. trachomatis* (Figure 6). In other bacteria, dedicated heat shock sigma factors of the RNA polymerase are responsible for the activation of numerous heat shock genes (Neidhardt and VanBogelen, 1981; Yamamori and Yura, 1982; Grossman et al., 1984; Völker et al., 1994; Yura, 1996; Hughes and Mathee, 1998). Interestingly, although *C. trachomatis* does not encode a clearly established heat shock sigma factor, our RNA-Seq data revealed a heat shock-induced 1.85-fold increase of *fliA* encoding σ^28^, and a concurrent 3.45-fold reduction of *rpoN* encoding σ^54^ (Supplementary Table S1). In addition, RNA-Seq also detected a statistically significant 1.45-fold increase in the RNA of *rpoD* encoding the housekeeping σ^66^. Consistent with the RNA-Seq analyses, qRT-PCR analysis detected 3.12- and 2.14-fold increases for *fliA* and *rpoD*, respectively, and a 1.56-fold decrease for *rpoN* (Figure 7A). These findings thus suggest that the relative up- or downregulation of all three sigma factors is an important determinant in facilitating the appropriate transcriptional response following heat shock.

In addition to *hrcA*, two other genes encoding transcription factors showed increased expression following heat shock. Transcripts of *ctl0478*, which encodes a Crp family transcriptional regulator, increased by 1.52-fold (Supplementary Table S1). This observation is consistent with the role of Crp as a transcriptional regulator of stress response in free-living bacteria (Kallipolitis and Valentin-Hansen, 1998; Ma et al., 2003; Shimizu, 2013). Moreover, transcripts of *euo*, which encodes a transcriptional repressor of late genes (Rosario and Tan, 2012; Rosario et al., 2014), showed a 1.53-fold expression increase in response to heat shock. Consistent with RNA-Seq, qRT-PCR detected a 3.18-fold increase in *euo* expression (Figure 7B). However, Euo target genes (e.g., *omcA*) did not show corresponding expression decreases following heat shock. The possible mechanism for these seemingly inconsistent observations will be discussed below.

RNA-Seq detected a significant 1.55-fold increase in the expression of *atoS*, a sensor histidine kinase, following heat shock that was confirmed by qRT-PCR (Supplementary Table S1). qRT-PCR additionally revealed a 2.1-fold increase in the expression of *atoC*, which encodes a σ^54^ RNAP transcriptional activator (Figure 7C). Together, AtoS and AtoC constitute a two-component system that regulates the σ^54^ RNA polymerase (Koo and Stephens, 2003; Soules et al., 2020). The significance of the upregulation of this transcriptional regulatory system and concurrent downregulated expression of *rpoN* (σ^54^) will also be discussed below.

Remarkably, RNA-Seq detected a heat shock-induced 2.5-fold decrease in *nusA*, encoding the transcription termination/antitermination protein NusA, and a 2.24-fold reduction in *rho*, encoding the transcription termination factor Rho (Supplementary Table S2). Furthermore, RNA-Seq detected significant downregulated expression of *ctl0818* (ATP-dependent helicase) and *ctl0463* (putative transcriptional regulator of the AlgH/UPF0301 family; Supplementary Table S2). Coupled with our detection of altered sigma factor-encoding gene expression, these findings suggest that alterations in transcription initiation, elongation, and termination all contribute to the heat shock-induced changes in the *C. trachomatis* transcriptome.

### Heat Shock-Upregulated Genes Have σ^28^ Promoters

As mentioned above, both RNA-Seq and qRT-PCR showed a heat shock-induced increase in σ^28^ (Figure 7A; Supplementary Table S1). To further investigate the role of σ^28^ in heat shock response, we parsed the RNA-Seq data for previously identified σ^28^ target genes (Yu et al., 2006b). Interestingly, among the 17 known σ^28^ targets in *C. trachomatis*, 6 (*pgk, trxB, dnaK, ctl0303, ctl0684*, and *ctl0815*) were found to be upregulated following heat shock, whereas 2 (*obgE* and *ctl0895*) were downregulated. These results suggest that upregulation of σ^28^ in response to heat shock in turn mediates the expression of a subset of its known target genes.

We next performed our own σ^28^ promoter search within the *C. trachomatis* genome. Using the consensus σ^28^ promoter binding sequence identified by Yu et al. (2006a; TAAAGWWY-N11/12-RYCGAWRN; where W is A/T, R is a purine, Y is a pyrimidine, and N is any nucleotide), motif-based sequence analyses identified 33 potential σ^28^ binding sites within 500 bps upstream of the ATG initiation codon among the 151 heat shock-upregulated genes. qRT-PCR was carried out for 15 of the 33 genes and confirmed their expression was increased in *C. trachomatis* cells upon heat shock (Table 2). In sum, our findings are consistent with the notion that σ^28^ plays an important functional role in mediating heat shock-induced gene expression.

**Table 2:** Heat shock-upregulated genes associated with a putative σ^28^ promoter.

Gene name	Locus	Description	TAAAGWWY-N11/12-RYCGAWRN	Distance (nt) to start codon	Fold change (RNA-Seq)	Fold change (qRT-PCR)	*p* (qRT-PCR)
*ctl003*	CTL0003	Putative T3SS chaperone	TAAAGTAC-N11-tTtGATGT	331	1.54	1.92 ± 0.24	5.74E-03
*sctQ*	CTL0041	T3S basal body	TgtAGAAT-N11-GCCGATAT	421	1.66	3.60 ± 0.30	2.96E-04
pgk*	CTL0062	Phosphoglycerate kinase	TtgAGTTT-N12-GCCtATAA	28	2.57	3.45 ± 0.27	2.52E-04
*ctl0064*	CTL0064	Hypothetical protein	TttAGATT-N11-ATCGATGC	297	1.61	nd^*^	na^**^
*birA*	CTL0094	Biotin--protein ligase	TAAAaAAT-N11-ATaGAAAG	262	1.54	nd	na
*ribH*	CTL0101	6,7-dimethyl-8-ribityllumazine synthase	TcAAGAAC-N11-ACCGcTGT	116	1.63	nd	na
*ctl0102*	CTL0102	Hypothetical protein	TAAAcTTT-N11-GCCaAAAT	370	1.51	1.76 ± 0.30	2.61E-02
*ftsK*	CTL0108	DNA translocase FtsK	aAAAGAAT-N12-ATCGAAGA	398	1.52	2.47 ± 0.36	5.89E-03
*murF*	CTL0125	UDP-N-acetylmuramoyl-tripeptide--D-alanyl-D-alanine ligase	TtAAGAAg-N12-ATCGAAcC	348	2.08	2.67 ± 0.50	9.46E-03
*miaA*	CTL0135	tRNA dimethylallyltransferase	aAAAGAAT-N11-ATaGAAAA	430	1.69	nd	na
*ctl0252*	CTL0252	Hypothetical protein	TAAAGATT-N11-ATaGAgGT	91	2.32	1.68 ± 0.24	5.19E-02
*pmpI*	CTL0254	Outer membrane protein PmpI	TAAAaATT-N12-ATCGATAA	204	2.67	1.72 ± 0.20	1.59E-02
*dcd*	CTL0294	Deoxycytidine triphosphate deaminase	TAAAGTcT-N12-ATaGATAA	19	1.85	nd	na
*ruvB*	CTL0296	Holliday junction DNA helicase RuvB	TAAAGTcT-N12-ATaGATAA	233	1.60	nd	na
*ssb*	CTL0300	Single-stranded DNA-binding protein	TAgAGTAT-N11-ACCaAAAA	137	2.45	3.07 ± 0.57	8.45E-03
ctl0303*	CTL0303	DNA polymerase, delta subunit	TtttGTAT-N11-GTCGAAAT	68	2.16	nd	na
*ctl0338*	CTL0338	Putative T3SS effector	TAAAGATC-N12-ATCacTGA	232	1.54	2.46 ± 0.39	5.82E-03
trxB*	CTL0354	Thioredoxin reductase	TttAGTTT-N12-GTCGAAAC	76	1.66	nd	na
*araD*	CTL0376	Ribulose-phosphate 3-epimerase	TAAAaTTT-N11-ATtGAAGT	42	1.61	nd	na
*ctl0399*	CTL0399	T3SS exported membrane protein	TcAAtATT-N12-ATCGATAA	393	2.18	3.04 ± 0.75	1.92E-02
*ctl0417a*	CTL0417a	Hypothetical protein	cAAAGAAC-N11-GCaGATGC	472	1.75	nd	na
*ihfA*	CTL0519	DNA-binding protein HU	TAAAGAgC-N11-tTaGATGC	337	2.43	3.49 ± 0.46	1.91E-03
*ctl0561*	CTL0561	Hypothetical protein	TAAAcAAC-N12-GCCaAAGA	134	1.84	nd	na
*atpE*	CTL0562	V-type ATP synthase subunit E	TAtAGTTT-N11-GCaGAAGG	152	1.63	3.27 ± 0.79	2.12E-02
*ctl0599*	CTL0599	Hypothetical protein	TAAgGTAT-N11-GTCGtAAT	172	1.61	nd	na
dnaK*	CTL0652	Chaperone protein dnaK	TAAAGgAA-N11-AaCGAAGA	35	3.75	5.99 ± 0.60	9.08E-04
ctl0684*	CTL0684	Hypothetical protein	TAAAGgAC-N10-cTCGAAC	16	1.53	nd	na
*euo*	CTL0706	Transcription repressor	TAcAGAcT-N12-ATCGAAAC	349	1.53	3.18 ± 0.46	2.50E-03
*ctl0791*	CTL0791	Putative membrane protein	TAAAaAAA-N12-ATCGAAGC	200	2.10	nd	na
*ctl0797*	CTL0797	Acyl-CoA thioesterase	TAAAGAAT-N12-ctCcATAC	471	1.88	nd	na
*ctl0808*	CTL0808	Hypothetical protein	gAAAGTAT-N11-cTCGAAAT	287	1.52	nd	na
ctl0815*	CTL0815	Lipoic acid ligase LplA1	TAAAGAgC-N13-cTCGAAGG	−92	2.58	nd	na
*ctl0870*	CTL0870	Hypothetical protein	cAAAGAAC-N12-GCCaATGG	61	2.04	nd	na

*TAAAGWWY-N11/12-RYCGAWRN, where W is A or T, R is a purine, Y is a pyrimidine, N is any nucleotide, and N11/12 is a spacer of 11 or 12 nucleotides (nt)*.

* *Not determined*.

** *Not applicable*.

### Most σ^54^ Target Genes Are Upregulated in the Presence of Downregulated *rpoN* and Upregulated *atoC* Following Heat Shock

The concurrent decreased expression of σ^54^ and increased expression of the σ^54^ RNAP activator AtoC (Figure 7; Supplementary Tables S1 and S2) suggests complexity in the regulated expression of σ^54^ target genes following heat shock. To investigate this apparent paradox more closely, we parsed the RNA-Seq data for the 13 known σ^54^ target genes recently identified by Soules et al. (2020). Interestingly, only three of the 13 σ^54^ target genes showed decreased RNA levels in *C. trachomatis* following heat shock, while the remaining 10 genes showed increased levels. We performed qRT-PCR analysis and confirmed the expression changes revealed by RNA-Seq (Table 3).

**Table 3:** Heat shock-induced σ^54^ target genes.

Gene name	Locus	Description	Fold change (RNA-Seq)	Fold change (qRT-PCR)	*p* (qRT-PCR)
*ctl0481*	CTL0481	Inclusion membrane protein	−2.16	−(1.73 ± 0.39)	6.08E-03
*sohB*	CTL0755	Protease	−2.05	−(1.33 ± 0.24)	1.18E-02
*polA*	CTL0754	DNA polymerase I	−1.69	−(1.41 ± 0.36)	1.05E-01
*ctl0003*	CTL0003	Putative T3SS chaperone	1.54	1.92 ± 0.24	5.74E-03
*ctl0260*	CTL0260	Putative membrane protein	1.88	2.64 ± 0.01	1.47E-04
*ctl0338*	CTL0338	Putative T3SS effector	1.54	2.07 ± 0.32	5.82E-03
*PLD*	CTL0339	Phosphatidylcholine-hydrolyzing phospholipase D (PLD) protein	1.84	2.46 ± 0.39	1.04E-02
*ctl0399*	CTL0399	T3SS exported membrane protein	2.18	3.04 ± 0.75	1.92E-02
*hrcA*	CTL0650	Heat-inducible transcriptional repressor	8.03	12.19 ± 2.27	4.54E-03
*grpE*	CTL0651	Heat shock protein-70 cofactor	3.44	4.58 ± 0.91	5.10E-03
*omcA*	CTL0703	Cysteine-rich outer membrane protein	1.54	2.63 ± 0.44	6.72E-03
*ctl0884*	CTL0884	Putative T3SS effector	1.6	1.91 ± 0.37	2.71E-02
*ctl0886*	CTL0886	T3SS effector; putative cell surface protein	1.58	2.12 ± 0.41	1.87E-02

Among the 10 σ^54^ target genes upregulated by heat shock are *hrcA* and *grpE*, which are on the same operon with tandem σ^66^–σ^54^ promoters (Tan et al., 1996; Soules et al., 2020). As shown in Figures 6A,B, expression of *hrcA* and *grpE* from the σ^66^ promoter is negatively regulated by HrcA and activated by heat shock. *omcA* is another heat shock-upregulated σ^54^ target gene with tandem σ^66^–σ^54^ promoters (Soules et al., 2020). Thus, decreased σ^54^ expression would allow for increased transcription from the σ^66^ promoters of the *hrcA-grpE* operon and *omcA* (Soules et al., 2020). Increased expression level of AtoC is likely responsible for the upregulated expression of the remaining seven genes lacking either a σ^66^ or σ^28^ promoter in response to heat shock (Soules et al., 2020). Taken together, the data presented in Figures 7A,C, and Table 3 suggest that concurrent downregulation of σ^54^ and upregulation of AtoC have differential effects on σ^54^ target gene expression. A hypothetical underlying regulatory mechanism will be presented below.

### Heat Shock Alters RNase Expression

RNA-Seq detected expression changes for several RNase-encoding genes following heat shock. Among them, *cafE* (RNase E) and *vacB* (RNase R) regulate mRNA decay in bacteria (Nilsson and Uhlin, 1991; Cheng and Deutscher, 2005; Mackie, 2013; Zhang and Gross, 2021). While *cafE* increased by 1.52-fold (Supplementary Table S1), *vacB* decreased by 1.87-fold (Supplementary Table S2). These data suggest that in addition to transcription, RNA decay also regulates *C. trachomatis* transcriptomic reprogramming in response to heat shock.

### *Chlamydia trachomatis* Heat Shock Transcriptional Regulatory Network

**Figure 8:**
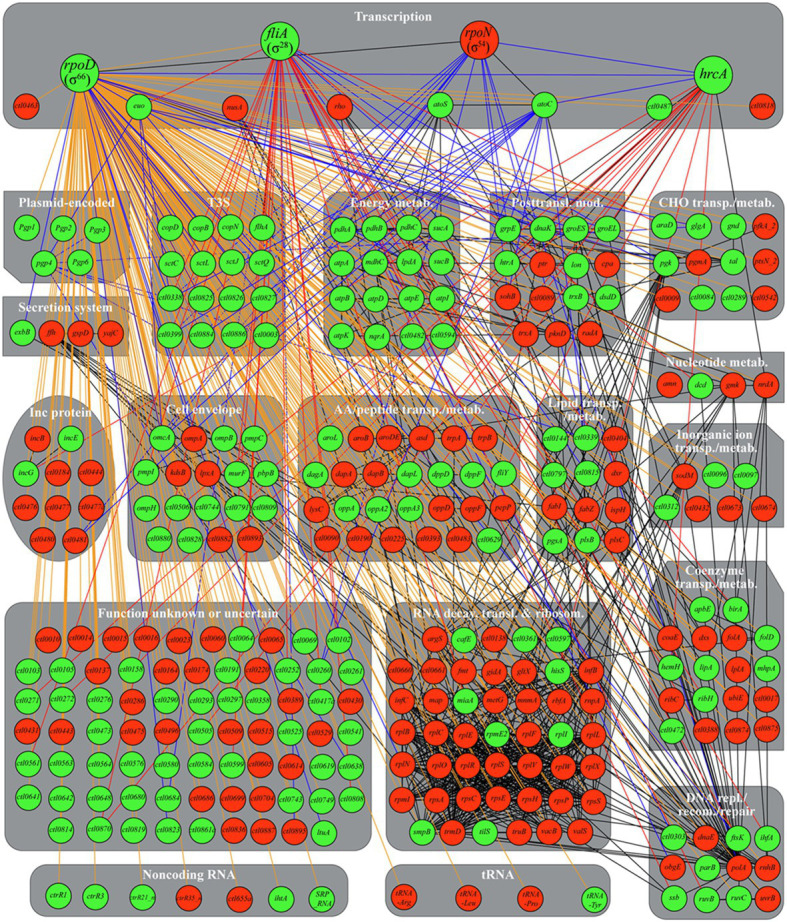
*Chlamydia trachomatis* heat shock transcriptional regulatory network. Green and red nodules signify heat-upregulated or downregulated genes, respectively, as determined by RNA-Seq and/or qRT-PCR (in cases of disagreements between the two analyses, qRT-PCR results were used to determine expression changes). Physical and/or functional associations identified by STRING are shown with black edges. σ^66^, σ^28^, σ^54^, HrcA, and Pgp4 target genes established in the literature but unrecognized by STRING are identified with blue edges. Putative σ^28^ and HrcA target genes identified in this study are identified with red edges. Genes without either a σ^28^ promoter or a σ^54^ promoter are treated as hypothetical σ^66^ targets and identified with orange edges. An interactive gephi file, which allows for clear viewing of individual associations, is presented as Supplementary Figure S1. Abbreviations: metab., metabolism; posttransl. mod., posttranslational modification; CHO, carbohydrate; transp., transport; Inc., inclusion membrane; AA, amino acid; transl., translation; ribosom., ribosomal structural and biogenesis; repl, replication; recom., recombination.

To provide a more systemic view of the heat shock-induced transcriptional reprogramming in *C. trachomatis*, we generated a *C. trachomatis* TRN diagram, in which red and green nodules represent heat-downregulated and upregulated genes, respectively (Figure 8). We used STRING to produce the heat shock TRN based on the program’s ability to integrate previously identified functional and structural association networks (Szklarczyk et al., 2018). We manually developed edges (using blue lines) for the three sigma factors based on previously reported target genes (Ricci et al., 1993, 1995; Fahr et al., 1995; Tan et al., 1996; Mathews and Timms, 2000; Shen et al., 2000, 2006; Yu and Tan, 2003; Yu et al., 2006b; Hefty and Stephens, 2007; Soules et al., 2020), and from Euo and Pgp4 to their known target genes (Rosario and Tan, 2012; Song et al., 2013; Rosario et al., 2014; Zhang et al., 2020). We also manually developed edges (using red lines) from *hrcA* and σ^28^ to their newly identified putative novel target genes (Figure 6; Table 2).

The addition of blue and red edges based on the literature and our own findings still left 251 heat shock-regulated genes without a sigma factor. We assume the remaining genes are σ^66^ targets given its “housekeeping” role in *C. trachomatis* (Engel and Ganem, 1990; Fahr et al., 1995; Douglas and Hatch, 2000) and manually developed edges to these genes using orange lines. An interactive Gephi Project file (Bastian et al., 2009) for this regulatory network can be accessed at the Figshare public data repository.^1^

The heat shock TRN shows that σ^66^ plays the most prominent role in reprogramming the transcriptome based on the number of its target genes (Figure 8). Notably, the transcription of the *hrcA-grpE-dnaK* operon is controlled not only by σ^66^ but additionally by a CIRCE element recognized by HrcA. During heat shock, HrcA is denatured, leading to greater induction of chaperone genes (connected to HrcA *via* black lines) which help maintain the structure and function of key bacterial proteins. Heat-induced HrcA denaturation also likely results in the derepression of a number of genes (connected to HrcA *via* red lines) with a variety of additional novel cellular functions (see Figure 6). Pgp4, which functions as a transcriptional coactivator (Song et al., 2013; Patton et al., 2018; Zhang et al., 2020), also positively reenforces the expression increases of at least four of σ^66^ target genes.

σ^28^ and the σ^54^/AtoC complex also play major roles in the heat-induced transcriptomic reprogramming as indicated by the numbers of genes connected to these transcriptional regulators (Figure 8). In addition to the numerous genes upregulated by elevated σ^66^ and σ^28^ levels, many σ^66^ target genes are downregulated (Figure 8) thus implicating functional roles for other transcriptional regulators. Interestingly, STRING identified eight genes regulated by the elongation factor and termination regulator NusA (*infB*, *rbfA*, *rplL*, and *truB*), the transcription terminator Rho (*sohB* and *coaE*), or both NusA and Rho (*rpsA* and *polA*). Similar to the observed *nusA* and *rho* downregulation, these eight genes were likewise downregulated, thus suggesting that NusA and Rho are responsible for their expression. However, how *nusA* and *rho*, which are presumed σ^66^ target genes, are downregulated is unclear. Several additional putative transcriptional regulators (e.g., CTL0463, CTL0487, and CTL0818) also undergo expression changes in response to heat shock, yet the function of these transcription factors in *C. trachomatis* remains unclear. The potential mechanisms of differential σ^54^ target gene expression in response to heat shock are discussed below.

Expression changes of a number of heat shock-regulated genes likely involve multiple transcriptional regulators and sigma factors. For example, while a major tandem σ^66^–σ^54^ promoter is upstream of the *hrcA-grpE-dnaK* operon (Soules et al., 2020), an additional σ^28^ promoter is upstream of the *dnaK* open reading frame (Yu et al., 2006b). *Ctl0338* is also a target of both σ^66^ and σ^54^ (Soules et al., 2020) and is further regulated by both Euo and Pgp4 (Zhang et al., 2020).

*cafE* (RNase E) and *vacB* (RNase R), which regulate mRNA decay (Nilsson and Uhlin, 1991; Cheng and Deutscher, 2005; Mackie, 2013), are also expected to participate in the *C. trachomatis* transcriptome reprogramming during heat shock. In summary, the heat shock TRN incorporates novel transcriptional regulatory relations identified in this study as well as those reported in the literature. However, the roles of additional transcriptional regulators and RNases in the transcriptomic reprogramming in response to heat shock in *C. trachomatis* require further investigations.

## Discussion

In response to infections, the human body raises its core temperature to inhibit the growth of pathogens including chlamydiae (Luger, 1948; Qvigstad et al., 1982; Dan et al., 1987; Wu et al., 2000; Reinhold et al., 2008, 2012; Stoner and Cohen, 2015; Clemmons et al., 2019). In this report, we have demonstrated that *C. trachomatis* is capable of mounting a very robust heat shock response. Our findings have important implications for chlamydial physiology and pathogenesis.

### (Patho)Physiological Significance of Transcriptomic Response in Response to Heat Shock

The functions of the proteins up- and downregulated by heat shock support the notion that *C. trachomatis* reprograms its transcriptome to survive during fever. Among the most striking transcriptional reprogramming events in *C. trachomatis* following heat shock is the increased expression of energy production and conversion genes (Figures 2, 3; Supplementary Table S1) coupled with broad expression decreases in genes encoding ribosomal proteins and proteins with functions in ribosomal biogenesis (Figure 2; Table 1). Under normal growth conditions, the majority of ATP molecules in bacteria may be spent on protein synthesis (Tempest and Neijssel, 1984; Szaflarski and Nierhaus, 2007). Ribosomal proteins and proteins involved in ribosomal biogenesis represent the two most abundant groups of bacterial cytosolic proteins (Ishihama et al., 2008). Reduction in their synthesis would result in tremendous energy savings. This, coupled with increased ATP production, would help RBs meet the increased demands for ATP-dependent processes, including chaperone-mediated protein folding (Xu et al., 1997; Derre et al., 1999a; Ranson et al., 2006; Clare et al., 2012; Wu et al., 2012; Sarbeng et al., 2015), proteolytic degradation (Goldberg, 1990; Liu et al., 2015; Roncarati and Scarlato, 2017; Wood et al., 2019, 2020), and T3S (Lugert et al., 2004; Wilharm et al., 2007).

In general, the eukaryotic intracellular environment is considered hostile to microorganisms (Moulder, 1982). Secretion of chlamydial effector proteins, mostly through T3S, into host cells is thought to be a crucial mechanism for chlamydial adaption to an obligate intracellular life (Fields and Hackstadt, 2000; Subtil et al., 2000; Fields et al., 2003, 2005; Clifton et al., 2004; Hefty and Stephens, 2007; Jamison and Hackstadt, 2008; Hobolt-Pedersen et al., 2009; Markham et al., 2009; Spaeth et al., 2009; Lutter et al., 2010; Chen et al., 2014; Nans et al., 2015; Bugalhão and Mota, 2019). It is likely that heat shock makes the intracellular environment even less accommodating to chlamydiae. To survive, RBs need to secrete higher amounts of effectors to prevent it from becoming inhospitable. In response to heat shock, *C. trachomatis* upregulates genes encoding T3SS structural components and chaperones, as well as T3S effectors (Figures 2, 4; Supplementary Table S1). Among the specific T3S effectors upregulated by heat shock, the increased expression of CTL0884 and CTL0886 is particularly intriguing. CTL0886 relieves the inhibitory effect of host protein ATG16L1 on *C. trachomatis* inclusion expansion (79). However, the precise mechanisms by which CTL0884 contributes to invasion and growth remain unresolved. CTL0884 contains a DUF582 sequence which interacts with components of the ESCRT (Vromman et al., 2016). Some ESCRT complexes are required for the multivesicular body (MVB) pathway and cytokinesis (Babst, 2011; Schmidt and Teis, 2012). Others may be involved in *C. trachomatis* extrusion exit (Zuck and Hybiske, 2019). Although previous research showed that the host proteins interacting with DUF582 are dispensable for *C. trachomatis* infection and growth, it is possible that they play more prominent roles for chlamydial survival under stress conditions.

Studies performed on animals indicate that the chlamydial plasmid and its encoded Pgp3 are virulence determinants (Liu et al., 2014b; Zhong, 2017). In this study, we found that heat shock upregulates *pgp3* and *pgp4*, as well as three other plasmid-encoded genes required for the plasmid maintenance, suggesting that Pgp3 and Pgp4 are important for chlamydial survival under stress conditions. Consistent with a role in stress response, it was previously shown that Pgp3 facilitates acid tolerance as evidenced by the fact that, unlike the wild-type bacterium, *pgp3*-deficient *C. muridarum* are incapable of colonizing the murine gastrointestinal tract following oral inoculation (Zhong, 2017; Shao et al., 2018; Zhang et al., 2019). Although a role for *pgp4* in pathogenicity has not been directly tested in animals, *C. muridarum* with mutations in *glgA*, a Pgp4 target gene that is also upregulated by heat shock, is attenuated in pathogenicity in the upper genital tract (Hu et al., 2020). The findings here thus reveal a correlation between proteins previously implicated in chlamydial pathogenicity with *Chlamydia* survival under stress conditions.

### Mechanisms of Heat Shock-Induced Transcriptomic Reprogramming

Heat shock sigma factors, found in most free-living bacteria but not *Chlamydia*, are responsible for the positive transcriptional regulation of heat shock genes (Neidhardt and VanBogelen, 1981; Yamamori and Yura, 1982; Grossman et al., 1984; Völker et al., 1994; Yura, 1996; Hughes and Mathee, 1998). The observation of increased *rpoD* and *fliA* expression in *C. trachomatis* during heat shock suggests that σ^66^ and σ^28^ activate numerous genes in response to the stress. However, it is somewhat counterintuitive that transcript copies of numerous presumptive σ^66^ target genes as well as some σ^28^ target genes were downregulated in response to heat shock. Notably, in addition to HrcA, several other transcription factor-encoding genes also showed expression changes following heat shock. Those transcription factors likely fine-tunes the expression of σ^66^ and σ^28^ target genes. Furthermore, the increased RNase E expression plausibly mediates the downregulation of some σ^66^ and σ^28^ target genes.

*rpoN*, which encodes σ^54^, was named for its role in response to nitrogen starvation in *E. coli* (Hunt and Magasanik, 1985). Previous studies have reported increased σ^54^ RNAP activity in response to different types of stress including heat shock in some bacteria (Schmid and Lidstrom, 2002). Surprisingly, we found that heat shock significantly downregulates *rpoN* expression in *C. trachomatis*. To the best of our knowledge, this is the first documentation of *rpoN* downregulation in response to environmental stress in bacteria. Unlike the σ^66^ and σ^28^ RNAP holoenzymes, the σ^54^ RNAP holoenzyme requires transcriptional activators, such as AtoC, in *C. trachomatis* for its functional activity (Merrick, 1993; Ghosh et al., 2010; Soules et al., 2020). Interestingly, we show here that σ^54^ downregulation is accompanied by an upregulation of the *atoS*-*atoC* operon, leading to upregulation of most, but not all, σ^54^ target genes. We hypothesize that the concurrent downregulation of σ^54^ and upregulation of AtoC differentially regulate the expression of genes with σ^54^ promoters depending on the inherent promoter affinity for the σ^54^ RNAP and whether or not the genes additionally carry σ^66^ or σ^28^ promoters. For example, when σ^54^ is downregulated, “simple” low-affinity σ^54^ promoters would lose access to the σ^54^ RNAP leading to reduced transcription. Moreover, σ^54^ RNAP might also serve as a *de facto* transcriptional repressor for genes with tandem σ^66^–σ^54^ or σ^28^–σ^54^ promoters when the AtoC levels are rate-limiting. In this case, downregulation of σ^54^ would allow σ^66^ RNAP or σ^28^ RNAP to transcribe such genes. On the other hand, activation of “high-affinity” σ^54^ promoters would be more dependent on elevated AtoC levels provided that σ^54^ levels do not become so low, such as to prevent any access to the promoter.

AtoS, which functions as a sensor kinase, and AtoC constitute a two-component system that regulates transcription of σ^54^ target genes (Koo and Stephens, 2003; Soules et al., 2020). The functional role of AtoS in facilitating this process has yet to be defined. Although we have not compared the kinase activity of AtoS at normal culture temperatures vs. heat shock conditions, we speculate that AtoS might serve as a sensor of temperature (or possibly of a metabolite whose levels have risen or fallen) during heat shock.

HrcA is the most widely distributed stress-inducible transcriptional repressor in bacteria and has long been recognized to bind and regulate target genes with CIRCE elements in their promoters (Baldini et al., 1998; Narberhaus, 1999; Stewart et al., 2002; Wilson and Tan, 2002, 2004; Spohn et al., 2004; Wilson et al., 2005; Holmes et al., 2010; Hanson and Tan, 2015; Pepe et al., 2018; Roncarati and Scarlato, 2018; Roncarati et al., 2019). By performing chromatin immunoprecipitation, De Barsy et al. (2016) detected HrcA binding to promoters lacking inverted repeat sequences. In this study, we identified DNA sequences that match the revised CIRCE element upstream of 13 *C. trachomatis* genes. Importantly, all 13 genes were upregulated in response to heat shock, supporting the notion that they serve as novel HrcA regulatory targets. Work is currently underway in our lab to investigate their functional roles in *C. trachomatis* in response to environmental stress.

Euo functions as a repressor of late genes (Rosario and Tan, 2012; Rosario et al., 2014). Despite increased *euo* expression, transcript copies of Euo target genes were also increased in response to heat shock. It is possible that the increased *euo* mRNA did not translate to increased Euo protein. Alternatively, the transcripts of Euo target genes might be stabilized during heat shock. Incidentally, we observed heat shock-downregulated *vacB* expression. It is conceivable that RNase R, the protein product of *vacB*, controls the decay of the RNAs of Euo target genes in *C. trachomatis*, based on previous findings made from other bacteria (Cheng and Deutscher, 2005; Zhang and Gross, 2021).

In summary, we have shown that *C. trachomatis*, which encodes only three sigma factors and a limited number of transcription factors, is able to mount a robust heat shock response that involves an extensive reprogramming of its transcriptome. This reprogramming meets the cell’s demand for: (a) increased energy production, (b) increased communication with host cells, (c) conservation of resources for protein synthesis, and (d) retention of the fitness and virulence determinants of its plasmid. The reprogramming also enables the RB to replicate its genome at a reduced rate. We show that the reprogramming is accomplished through the actions of all three sigma factors, the heat-inducible transcriptional repressor HrcA, the plasmid-encoded transcription regulator Pgp4, the σ^54^ RNAP transcriptional activator AtoC, and additional yet-to-be-defined transcription regulators. Clearly, and despite its small size, *Chlamydia* is a sophisticated organism. Such sophistication likely has made *Chlamydia* a highly successful parasite. Future strategies that can specifically target and disrupt *Chlamydia*’s heat shock response will likely be of therapeutic value.

## Data Availability Statement

The datasets presented in this study can be found in online repositories. The names of the repository/repositories and accession number(s) can be found in the article/Supplementary Material.

## Author Contributions

YH, XW, and HF conceived and designed the project. YH, WW, BL, YW, and KW carried out the experiments. YH, WW, YZ, JDF, ZL, XW, and HF analyzed the data. YH, JDF, and HF wrote the manuscript. All authors contributed to the article and approved the submitted version.

## Funding

This work was supported in part by grants from the National Institutes of Health (grant nos. AI140167 and AI154305 to HF) and National Natural Sciences Foundation of China (grant nos. 82072306 and 81371834 to XW). Genome Sequencing Facility at UTHSA is supported by NIH-NCI P30 CA054174 (Cancer Center at UT Health San Antonio), NIH Shared Instrument grant OD021805 (S10 grant), and CPRIT Core Facility Award (RP160732). YH was supported by a scholarship (award no. 201806370171) from the China Scholarship Council (CSC) from September 2018 to September 2020.

## Conflict of Interest

The authors declare that the research was conducted in the absence of any commercial or financial relationships that could be construed as a potential conflict of interest.

## Publisher’s Note

All claims expressed in this article are solely those of the authors and do not necessarily represent those of their affiliated organizations, or those of the publisher, the editors and the reviewers. Any product that may be evaluated in this article, or claim that may be made by its manufacturer, is not guaranteed or endorsed by the publisher.

## Footnotes

^1^ https://figshare.com/s/120f3e12e8034a53e6ab
