# Supplementary material for: Robust Heat Shock Response in Chlamydia Lacking a Typical Heat Shock Sigma Factor
Source: Front Microbiol. 2022 Jan 3;12:812448. doi: 10.3389/fmicb.2021.812448 (PMC8762339; doi:10.3389/fmicb.2021.812448)
Supplement: Supplementary file 2 [file Data_Sheet_2.PDF]

Table S1. 151 heat shock-upregulated genes detected by RNA-seq (≥ 1.5-fold change, P < 0.005)

Functional class	Gene name	Locus	Description	Fold change
Energy production and conversion	*nqrA*	CTL0002	Na (+)-translocating NADH-quinone reductase subunit A	1.62
	sucA	CTL0310	2-oxoglutarate dehydrogenase subunit E1	2.05
	ctl0482	CTL0482	Na+/H+-dicarboxylate symporter (C)	1.84
	pdhA	CTL0497	Pyruvate dehydrogenase E1 component subunit alpha	1.71
	atpE	CTL0562	V-type ATP synthase subunit E	1.63
	ctl0594	CTL0594	2-oxoisovalerate dehydrogenase subunit alpha/beta	1.56
	mdhC	CTL0630	Malate dehydrogenase	1.68
Carbohydrate transport and metabolism	*pgk*	CTL0062	Phosphoglycerate kinase	2.57
	ctl0084	CTL0084	UDP-N-acetylglucosamine pyrophosphorylase	1.52
	ctl0289	CTL0289	Membrane transport/efflux protein	1.94
	gnd	CTL0319	6-phosphogluconate dehydrogenase	1.55
	araD	CTL0376	Ribulose-phosphate 3-epimerase	1.61
	tal	CTL0565	Transaldolase	1.65
Amino acid/peptide transport and metabolism	*dppF*	CTL0058	ABC transporter ATP-binding protein	1.96
	dppD	CTL0059	Oligopeptide ABC transporter ATP-binding protein	5.41
	dagA	CTL0104	Na+/alanine symporter	2.70
	oppA	CTL0394	Oligopeptide ABC transporter substrate-binding protein	2.20
	oppA2	CTL0427	Oligopeptide ABC transporter substrate-binding protein	1.96
	oppA3	CTL0450	Oligopeptide ABC transporter substrate-binding protein	2.12
	aroL	CTL0621	Shikimate kinase	1.85
	ctl0629	CTL0629	Putative oxidoreductase	1.74
	dapL	CTL0646	L, L-diaminopimelate aminotransferase	2.14
	fliY	CTL0747	ABC transporter substrate-binding protein	1.86
Lipid transport and metabolism	*ctl0144*	CTL0144	1-acyl-sn-glycerol-3-phosphate acyltransferase	1.51
	pgsA	CTL0166	CDP-diacylglycerol--glycerol-3-phosphate 3-phosphatidyltransferase	2.98
	plsB	CTL0176	Glycerol-3-phosphate acyltransferase	1.99
	ctl0339	CTL0339	Phosphatidylcholine hydrolyzing phospholipase D(PLD) protein	1.84
	ctl0797	CTL0797	Acyl-CoA thioesterase	1.88
	ctl0815	CTL0815	Lipoic acid ligase LplA1	2.58
Coenzyme transport and metabolism	*birA*	CTL0094	Biotin--protein ligase	1.54
	ribH	CTL0101	6,7-dimethyl-8-ribityllumazine synthase	1.63
	apbE	CTL0333	Thiamine biosynthesis lipoprotein	1.62
	folD	CTL0334	Bifunctional 5,10-methylene-tetrahydrofolate dehydrogenase/5,10-methylene-tetrahydrofolate cyclohydrolase	2.07
	mhpA	CTL0403	FAD-dependent monooxygenase	1.91
	ctl0472	CTL0472	Aromatic acid decarboxylase	1.59
	hemH	CTL0746	Ferrochelatase	1.84
	lipA	CTL0821	Lipoyl synthase	1.63
Inorganic ion transport and metabolism	*ctl0096*	CTL0096	Metal transporting ATPase	1.58
	ctl0097	CTL0097	Putative integral membrane protein	2.03
	ctl0312	CTL0312	Polyphenol oxidase	1.62
Nucleotide metabolism	*dcd*	CTL0294	Deoxycytidine triphosphate deaminase	1.85

DNA replication, recombination and repair	*parB*	CTL0057	Chromosome partitioning protein ParB	1.74
	ftsK	CTL0108	DNA translocase FtsK	1.52
	ruvB	CTL0296	Holliday junction DNA helicase RuvB	1.60
	ssb	CTL0300	Single-stranded DNA-binding protein	2.45
	ctl0303	CTL0303	DNA polymerase, delta subunit	2.16
	ihfA	CTL0519	DNA-binding protein HU	2.43
	ruvC	CTL0764	Crossover junction endodeoxyribonuclease RuvC	1.52
Transcription	*fliA*	CTL0317	RNA polymerase sigma factor sigma-28	1.85
	ctl0487	CTL0487	Crp family transcriptional regulator	1.52
	hrcA	CTL0650	Heat-inducible transcription repressor	8.03
	euo	CTL0706	Transcription repressor of late genes	1.53
	atoS	CTL0727	Two component system sensor histidine kinase	1.55
Translation, ribosomal structure and biogenesis	*miaA*	CTL0135	tRNA dimethylallyltransferase	1.69
	rplI	CTL0172	50S ribosomal protein L9	1.63
	cafE	CTL0177	Ribonuclease E	1.52
	tilS	CTL0212	tRNA (Ile)-lysidine synthase	2.09
	rpmE2	CTL0277	50S ribosomal protein L31 type B	2.26
	smpB	CTL0332	SsrA-binding protein	1.73
	ctl0361	CTL0361	tRNA pseudouridine synthase A	1.77
	ctl0597	CTL0597	O-sialoglycoprotein endopeptidase	1.53
	hisS	CTL0805	Histidine--tRNA ligase	1.70
tRNA	*tRNA-Tyr*	CTL_t35	tRNA-Tyr	1.95
Posttranslational modification,protein turnover, chaperones	*htrA*	CTL0195	Serine protease	2.68
	trxB	CTL0354	Thioredoxin reductase	1.66
	groEL	CTL0365	Chaperonin GroEL	2.29
	groES	CTL0366	Co-chaperonin GroES	2.35
	lon	CTL0598	Lon protease	2.12
	grpE	CTL0651	Heat shock protein-70 cofactor	3.44
	dnaK	CTL0652	Heat shock protein-70	3.75
	dsdD	CTL0859	Thiol:disulfide interchange protein	1.58
Cell wall/membrane/envelope biogenesis	*pbpB*	CTL0051	Penicillin-binding protein	1.60
	ompB	CTL0082	Outer membrane protein B	2.02
	murF	CTL0125	UDP-N-acetylmuramoyl-tripeptide--D-alanyl-D-alanine ligase	2.08
	pmpI	CTL0254	Outer membrane protein PmpI	2.67
	ompH	CTL0494	Outer membrane protein	1.70
	ctl0506	CTL0506	Inner membrane protein	2.17
	pmpC	CTL0671	Outer membrane protein PmpC	1.58
	omcA	CTL0703	Cysteine-rich outer membrane protein	1.54
	ctl0744	CTL0744	Putative membrane protein	2.19
	ctl0791	CTL0791	Putative membrane protein	2.10
	ctl0809	CTL0809	Putative exported protein	1.78
	ctl0828	CTL0828	Putative membrane protein	3.15
	ctl0880	CTL0880	Putative integral membrane protein	1.72
Inclusion membrane protein	*incG*	CTL0373	Inclusion membrane protein G	2.03
	incE	CTL0371	Inclusion membrane protein E	1.57

T3SS secretion system protein	*ctl0003*	CTL0003	Putative T3SS chaperone	1.54
	sctQ	CTL0041	T3S component, basal body	1.66
	sctC	CTL0043	T3S structural protein (outer membrane ring)	1.58
	flhA	CTL0316	Flagellar biosynthesis protein (export)	1.59
	ctl0338	CTL0338	Putative T3SS effector	1.54
	copN	CTL0344	Low calcium response protein E (T3SS effector protein)	1.76
	ctl0399	CTL0399	T3SS exported membrane protein	2.18
	sctJ	CTL0822	T3SS protein, membrane component	1.98
	sctL	CTL0824	T3SS protein	1.80
	copB	CTL0841	T3SS translocator subunit CopB	1.90
	ctl0884	CTL0884	Putative T3SS effector	1.60
	ctl0886	CTL0886	PutativeT3SS effector; putative cell surface protein	1.58
Secretion, and vesicular transport	*exbB*	CTL0860	Biopolymer transport protein	2.10
Function unknown and uncertain	*ctl0064*	CTL0064	Hypothetical protein	1.61
	ctl0069	CTL0069	Hypothetical protein	1.52
	ctl0102	CTL0102	Hypothetical protein	1.51
	ctl0103	CTL0103	Hypothetical protein	1.85
	ctl0105	CTL0105	Hypothetical protein	1.98
	ctl0158	CTL0158	Hypothetical protein	1.54
	ctl0191	CTL0191	Hypothetical protein	2.27
	ctl0252	CTL0252	Hypothetical protein	2.32
	ctl0260	CTL0260	Hypothetical protein	1.88
	ctl0261	CTL0261	Hypothetical protein	1.50
	ctl0271	CTL0271	Hypothetical protein	5.40
	ctl0272	CTL0272	Hypothetical protein	2.96
	ctl0276	CTL0276	Hypothetical protein	3.88
	ctl0290	CTL0290	Hypothetical protein	2.93
	ctl0293	CTL0293	Hypothetical protein	2.18
	ctl0297	CTL0297	Hypothetical protein	2.59
	ctl0358	CTL0358	Hypothetical protein	1.55
	ctl0417a	CTL0417a	Hypothetical protein	1.75
	ctl0473	CTL0473	Hypothetical protein	1.97
	ctl0525	CTL0525	Hypothetical protein	1.63
	ctl0541	CTL0541	Hypothetical protein	1.58
	ctl0561	CTL0561	Hypothetical protein	1.84
	ctl0563	CTL0563	Hypothetical protein	2.18
	ctl0564	CTL0564	Hypothetical protein	1.50
	ctl0576	CTL0576	Hypothetical protein	1.99
	ctl0580	CTL0580	Hypothetical protein	2.34
	ctl0584	CTL0584	Hypothetical protein	1.94
	ctl0599	CTL0599	Hypothetical protein	1.61
	ctl0619	CTL0619	Hypothetical protein	1.55
	ltuA	CTL0631	Late transcription unit A protein	1.81
	ctl0638	CTL0638	Hypothetical protein	3.69
	ctl0641	CTL0641	Hypothetical protein	2.50
	ctl0642	CTL0642	Hypothetical protein	1.78

Function unknown and uncertain	*ctl0648*	CTL0648	Hypothetical protein	1.75
	ctl0680	CTL0680	Hypothetical protein	1.55
	ctl0684	CTL0684	Hypothetical protein	1.53
	ctl0743	CTL0743	Hypothetical protein	1.59
	ctl0749	CTL0749	Hypothetical protein	1.83
	ctl0808	CTL0808	Hypothetical protein	1.52
	ctl0814	CTL0814	Hypothetical protein	2.37
	ctl0819	CTL0819	Hypothetical protein	2.66
	ctl0861a	CTL0861A	Hypothetical protein	1.74
	ctl0870	CTL0870	Hypothetical protein	2.04
ncRNA	ctrR1	ctrR1	ncRNA	1.85
	ctrR21_n	ctrR21_n	ncRNA	1.52
	ctrR3	ctrR3	ncRNA	2.04
	ihtA	ihtA	ncRNA	3.00
	SRP_RNA	SRP_RNA	ncRNA	2.45
Plasmid encode gene	*Pgp1*	pL2-03	Plasmid maintenance, virulence plasmid helicase	2.44
	Pgp2	pL2-04	Plasmid maintenance	1.67
	Pgp3	pL2-05	Virulence factor	1.78
	Pgp6	pL2-08	Plasmid maintenance	1.60
