# Supplementary material for: Robust Heat Shock Response in Chlamydia Lacking a Typical Heat Shock Sigma Factor
Source: Front Microbiol. 2022 Jan 3;12:812448. doi: 10.3389/fmicb.2021.812448 (PMC8762339; doi:10.3389/fmicb.2021.812448)
Supplement: Supplementary file 3 [file Data_Sheet_3.PDF]

Table S2. 152 heat shock-downregulated genes detected by RNA-seq (≥ 1.5-fold change, P < 0.005)

Functional class	Gene name	Locus	Description	Fold change
Carbohydrate transport and metabolism	*ctl0009*	CTL0009	Membrane efflux protein	-3.38
	pgmA	CTL0091	2,3-bisphosphoglycerate-dependent phosphoglycerate mutase	-1.52
	pfkA_2	CTL0459	Pyrophosphate--fructose 6-phosphate 1-phosphotransferase	-1.55
	ctl0542	CTL0542	PTS fructose transporter subunit IIA	-1.66
	ptsN_2	CTL0543	PTS fructose transporter subunit IIA	-1.91
Amino acid/peptide transport and metabolism	*ctl0090*	CTL0090	Aminotransferase	-1.87
	ctl0190	CTL0190	Tyrosine-specific transport protein	-1.73
	ctl0225	CTL0225	Putative integral membrane protein	-1.93
	ctl0393	CTL0393	Microsomal dipeptidase	-1.64
	trpB	CTL0423	Tryptophan synthase subunit beta	-4.64
	trpA	CTL0424	Tryptophan synthase subunit alpha	-2.50
	oppD	CTL0453	Oligopeptide ABC transporter ATP-binding protein	-3.11
	oppF	CTL0454	Oligopeptide ABC transporter ATP-binding protein	-2.77
	ctl0483	CTL0483	Sodium-dependent amino acid transporter	-1.63
	dapA	CTL0615	4-hydroxy-tetrahydrodipicolinate synthase	-3.01
	lysC	CTL0616	Aspartokinase	-2.24
	asd	CTL0617	Aspartate-semialdehyde dehydrogenase	-2.21
	dapB	CTL0618	4-hydroxy-tetrahydrodipicolinate reductase	-2.35
	aroB	CTL0623	3-dehydroquinate synthase	-1.55
	aroDE	CTL0624	Bifunctional 3-dehydroquinate dehydratase/shikimate dehydrogenase	-1.55
	pepP	CTL0837	Aminopeptidase	-1.66
Lipid transport and metabolism	*ispH*	CTL0234	4-hydroxy-3-methylbut-2-enyl diphosphate reductase	-3.94
	dxr	CTL0327	1-deoxy-D-xylulose 5-phosphate reductoisomerase	-1.56
	fabI	CTL0359	Enoyl-(acyl carrier protein) reductase	-1.57
	ctl0404	CTL0404	Lysophospholipase, alpha-beta hydrolase superfamily (I)	-2.14
	plsC	CTL0713	1-acyl-sn-glycerol-3-phosphate acyltransferase	-2.23
	fabZ	CTL0794	3-hydroxyacyl-ACP dehydratase	-1.67
Coenzyme transport and metabolism	*ctl0017*	CTL0017	5-formyltetrahydrofolate cyclo-ligase	-2.73
	ctl0388	CTL0388	Methyltransferase	-1.50
	dxs	CTL0585	1-deoxy-D-xylulose-5-phosphate synthase	-1.61
	ribC	CTL0662	Riboflavin synthase	-2.52
	ubiE	CTL0687	Demethylmenaquinone methyltransferase	-1.73
	coaE	CTL0753	Dephospho-CoA kinase	-2.02
	lplA	CTL0761	Lipoate-protein ligase A	-1.76
	ctl0874	CTL0874	Pyrroloquinoline quinone (PQQ) biosynthesis protein C	-1.59
	ctl0875	CTL0875	Gamma-glutamyl ligase	-3.78
	folA	CTL0876	Dihydrofolate reductase	-2.84
Inorganic ion transport and metabolism	*ctl0432*	CTL0432	ABC transporter ATP-binding protein	-1.83
	sodM	CTL0546	Superoxide dismutase	-1.59
	ctl0673	CTL0673	Metal ABC transporter ATP-binding protein	-1.54
	ctl0674	CTL0674	Metal ABC transporter permease	-2.38
Nucleotide metabolism	*amn*	CTL0120	AMP nucleosidase	-1.57
	nrdA	CTL0199	Ribonucleoside-diphosphate reductase subunit alpha	-2.13
	gmk	CTL0285	Guanylate kinase	-2.19

DNA replication, recombination and repair	*rnhB*	CTL0284	Ribonuclease HII	-2.24
	polA	CTL0754	DNA polymerase I	-1.69
	dnaE	CTL0807	DNA polymerase III subunit alpha	-1.65
	uvrB	CTL0849	Excinuclease ABC subunit B	-2.00
	obgE	CTL0675	GTPase ObgE	-2.58
Transcription	*nusA*	CTL0352	Transcription termination/antitermination protein NusA	-2.50
	ctl0463	CTL0463	Putative transcriptional regulator, AlgH/UPF0301 family	-1.53
	rpoC	CTL0566	DNA-directed RNA polymerase subunit beta'	-1.52
	rpoB	CTL0567	DNA-directed RNA polymerase subunit beta	-1.91
	rho	CTL0752	Transcription termination factor	-2.24
	ctl0818	CTL0818	Helicase	-2.30
	rpoN	CTL0873	RNA polymerase sigma-54 factor	-3.40
Translation, ribosomal structure and biogenesis	*ctl0138*	CTL0138	Ribosomal silencing factor RsfS, regulates association of 30S and 50S subunits	-1.59
	rnpA	CTL0153	Ribonuclease P protein component	-1.65
	infC	CTL0205	Translation initiation factor IF-3	-1.51
	rpmI	CTL0206	50S ribosomal protein L35	-1.60
	map	CTL0224	Methionine aminopeptidase	-1.73
	rpsP	CTL0281	30S ribosomal protein S16	-2.38
	trmD	CTL0282	Fused tRNA (guanine-N(1)-)-methyltransferase/hypothetical protein	-2.20
	rplS	CTL0283	50S ribosomal protein L19	-2.37
	metG	CTL0287	Methionine--tRNA ligase	-2.19
	truB	CTL0349	tRNA pseudouridine synthase B	-1.77
	rbfA	CTL0350	Ribosome-binding factor A	-1.89
	infB	CTL0351	Translation initiation factor IF-2	-1.98
	rpsA	CTL0353	30S ribosomal protein S1	-2.50
	mnmA	CTL0539	tRNA-specific 2-thiouridylase MnmA	-1.51
	valS	CTL0554	Valine--tRNA ligase	-2.19
	rplL	CTL0568	50S ribosomal protein L7/L12	-1.59
	tufA	CTL0574	Elongation factor Tu	-1.77
	vacB	CTL0654	Ribonuclease R	-1.87
	ctl0660	CTL0660	SpoU family rRNA methylase	-2.19
	ctl0661	CTL0661	SAM-dependent methyltransferase	-2.17
	gltX	CTL0705	Glutamate--tRNA ligase	-1.57
	argS	CTL0714	Arginine--tRNA ligase	-2.46
	gidA	CTL0760	tRNA uridine 5-carboxymethylaminomethyl modification protein GidA	-1.59
	rplO	CTL0773	50S ribosomal protein L15	-1.81
	rpsE	CTL0774	30S ribosomal protein S5	-1.60
	rplR	CTL0775	50S ribosomal protein L18	-1.65
	rplF	CTL0776	50S ribosomal protein L6	-1.64
	rpsH	CTL0777	30S ribosomal protein S8	-1.71
	rplE	CTL0778	50S ribosomal protein L5	-1.78
	rplX	CTL0779	50S ribosomal protein L24	-1.67
	rplN	CTL0780	50S ribosomal protein L14	-1.55
	rpsC	CTL0784	30S ribosomal protein S3	-1.52
	rplV	CTL0785	50S ribosomal protein L22	-1.80
	rpsS	CTL0786	30S ribosomal protein S19	-1.52

Translation, ribosomal structure and biogenesis	*rplB*	CTL0787	50S ribosomal protein L2	-1.53
	rplW	CTL0788	50S ribosomal protein L23	-1.69
	rplD	CTL0789	50S ribosomal protein L4	-2.06
	rplC	CTL0790	50S ribosomal protein L3	-1.68
	fmt	CTL0792	Methionyl-tRNA formyltransferase	-2.55
tRNA	*tRNA-Leu*	CTL_t17	tRNA-Leu	-1.70
	tRNA-Arg	CTL_t05	tRNA-Arg	-1.65
	tRNA-Pro	CTL_t25	tRNA-Pro	-1.53
Posttranslational modification, protein turnover, chaperones	*ctl0089*	CTL0089	NifU homolog involved in Fe-S cluster formation	-2.74
	ptr	CTL0175	Insulinase/protease	-2.16
	cpa	CTL0233	Putative exported protease	-1.56
	radA	CTL0550	DNA repair protein RadA	-1.71
	pknD	CTL0553	Serine/threonine-protein kinase	-1.71
	sohB	CTL0755	Protease	-2.05
	trxA	CTL0801	Negative regulator of GroEL, contains thioredoxin-like and TPR-like domains	-1.51
Cell wall/membrane/ envelope biogenesis	*ompA*	CTL0050	Major outer membrane porin	-3.15
	kdsB	CTL0434	3-deoxy-manno-octulosonate cytidylyltransferase	-2.30
	lpxA	CTL0793	Acyl-[acyl-carrier-protein]--UDP-N-acetylglucosamine O-acyltransferase	-2.11
	ctl0882	CTL0882	Putative membrane protein	-2.33
	ctl0893	CTL0893	Glucosamine-1-phosphate acetyltransferase	-1.80
Inclusion membrane protein	*ctl0184*	CTL0184	Inclusion membrane protein	-1.78
	ctl0444	CTL0444	Inclusion membrane protein	-1.70
	ctl0476	CTL0476	Inclusion membrane protein	-2.34
	ctl0477	CTL0477	Inclusion membrane protein	-1.82
	ctl0477a	CTL0477A	Inclusion membrane protein	-1.99
	ctl0480	CTL0480	Inclusion membrane protein	-1.89
	ctl0481	CTL0481	Inclusion membrane protein	-2.16
	incB	CTL0484	Inclusion membrane protein B	-1.80
Secretion, and vesicular transport	*yajC*	CTL0110	Preprotein translocase subunit YajC	-2.77
	ffh	CTL0280	Signal recognition particle subunit FFH	-1.70
	gspD	CTL0835	Type II secretion system protein D	-1.73
Function unknown or uncertain	*ctl0010*	CTL0010	Hypothetical protein	-1.77
	ctl0014	CTL0014	Hypothetical protein	-2.81
	ctl0015	CTL0015	Hypothetical protein	-2.44
	ctl0016	CTL0016	Hypothetical protein	-3.74
	ctl0023	CTL0023	Hypothetical protein	-1.56
	ctl0060	CTL0060	Hypothetical protein	-1.82
	ctl0065	CTL0065	Hypothetical protein	-1.52
	ctl0137	CTL0137	Hypothetical protein	-1.83
	ctl0164	CTL0164	Hypothetical protein	-1.58
	ctl0174	CTL0174	Hypothetical protein	-2.08
	ctl0220	CTL0220	Hypothetical protein	-1.79
	ctl0286	CTL0286	Hypothetical protein	-2.70
	ctl0389	CTL0389	Hypothetical protein	-2.21
	ctl0430	CTL0430	Hypothetical protein	-1.51
	ctl0431	CTL0431	Hypothetical protein	-2.43
	ctl0443	CTL0443	Hypothetical protein	-1.66
	ctl0475	CTL0475	Hypothetical protein	-2.15

Function unknown or uncertain	*ctl0496*	CTL0496	Hypothetical protein	-1.88
	ctl0509	CTL0509	Hypothetical protein	-1.76
	ctl0515	CTL0515	Hypothetical protein	-1.51
	ctl0529	CTL0529	Hypothetical protein	-1.69
	ctl0605	CTL0605	Hypothetical protein	-1.69
	ctl0614	CTL0614	Hypothetical protein	-1.92
	ctl0686	CTL0686	Hypothetical protein	-1.62
	ctl0699	CTL0699	Hypothetical protein	-1.87
	ctl0704	CTL0704	Hypothetical protein	-1.55
	ctl0836	CTL0836	Hypothetical protein	-1.93
	ctl0887	CTL0887	Hypothetical protein	-2.27
	ctl0895	CTL0895	Hypothetical protein	-1.65
ncRNA	CTL655a	CTL655a	ncRNA	-2.48
	ctrR35_n	ctrR35_n	ncRNA	-1.52
